# Metastatic Pheochromocytoma/Paraganglioma: Diagnostic Performance of Functional Imaging (^18^F-Fluoro-L-DOPA-, ^68^Ga-DOTA- and ^18^F-Fluoro-Deoxyglucose-Based PET/CT) and of ^123^I-MIBG Scintigraphy in 57 Patients and 527 Controls During Long-Term Follow-Up

**DOI:** 10.3390/cancers17233855

**Published:** 2025-11-30

**Authors:** Andreas Scheuba, Oana Cristina Kulterer, Reinhard Lehner, Mateja Rybiczka-Tešulov, Harald Esterbauer, Wolfgang Raber

**Affiliations:** 1 Department of General Surgery, Medical University of Vienna, University Hospital Vienna, 1090 Vienna, Austria; andreas.scheuba@meduniwien.ac.at; 2 Department of Biomedical Imaging and Image-Guided Therapy, Division of Nuclear Medicine, Medical University of Vienna, University Hospital Vienna, 1090 Vienna, Austria; oana.kulterer@meduniwien.ac.at; 3 Department of Medical Genetics, Medical University of Vienna, University Hospital Vienna, 1090 Vienna, Austria; reinhard.lehner@meduniwien.ac.at (R.L.); mateja.rybiczka-tesulov@meduniwien.ac.at (M.R.-T.); 4 Department of Laboratory Medicine, Medical University of Vienna, University Hospital Vienna, 1090 Vienna, Austria; harald.esterbauer@meduniwien.ac.at; 5 Department of Medicine III, Clinical Division of Endocrinology and Metabolism, Medical University of Vienna (MUW), University Hospital Vienna (AKH), Währinger Gürtel 18-20, 1090 Vienna, Austria

**Keywords:** pheochromocytoma, paraganglioma, radionuclide imaging, positron emission tomography, F-DOPA, DOTANOC, DOTATOC, FDG, MIBG, long-term follow-up

## Abstract

**Background**: Large-scale data on the diagnostic performance of functional imaging in metastatic pheochromocytoma/paraganglioma (PPGL) are scarce. **Objective**: To analyze the diagnostic accuracy of functional imaging for the assessment of metastases during long-term follow-up (FU). **Design**: Retrospective cohort study, 1991–2025. **Setting**: Referral center. **Outcomes**: Sensitivity and specificity of ^123^MIBG-, ^18^F-DOPA-, ^68^GaDOTA-based and ^18^FDG PET/CT. **Patients:** Patients with metastatic PPGL and without PPGL, ≥1 functional imaging prior to first diagnosis and/or during FU and FU ≥ 3 months. **Results**: 59 ^123^MIBG-, 101 ^18^F-DOPA-, 11 ^18^FDG- and 74 ^68^GaDOTA-based PET/CT were performed in 57 patients with metastatic PPGL and 37 ^123^MIBG-, 323 ^18^F-DOPA-, 259 ^18^FDG- and 641 ^68^GaDOTA-based imaging in 527 patients without PPGL. FU was 11.6 ± 11.4 and 5.1 ± 4.7 years, respectively. Sensitivity for the detection of all metastases (total cohort) by patient-based analysis was comparable between ^18^F-DOPA (77%), both ^68^GaDOTA-based tracers combined (67%) and ^123^MIBG (72%); lesion-based analysis was better for ^18^F-DOPA (94%) than for ^68^GaDOTA (85%) and ^123^MIBG (67%). Specificity (patient- and lesion-based) of ^18^F-DOPA vs. ^68^GaDOTA (96–99%) was comparable and better than ^123^MIBG (73%) and ^18^FDG (75%). **Conclusions**: Sensitivity of ^18^F-DOPA was superior to ^68^GaDOTA for the detection of all and bone metastases in the total cohort and in patients with PCC, that of ^68^GaDOTA was better for bone metastases with PGL, specificity was comparable and for both was better than for ^18^FDG and ^123^MIBG. Given the beneficial pharmaceutical properties and favorable diagnostic performance, ^18^F-DOPA may be a good alternative to ^68^GaDOTA-based tracers for the functional imaging of metastatic PPGL.

## Simple Summary

Metastases develop in about 10–20% of patients with pheochromocytoma (PCC)/paraganglioma (PGL), together with PPGL, and represent the main risk factor for mortality. Functional imaging with PET/CT has revolutionized the detection of PPGL; however, its diagnostic performance has only been investigated in small samples of patients with metastatic PPGL. We studied 57 patients with metastatic PPGL and 527 without PPGL for >11 and >5 years, respectively. The main findings may be considered the superior sensitivity of F-DOPA vs. GaDOTA for the detection of all and bone metastases in the total cohort and in patients with PCC, while sensitivity to detect bone metastases in patients with PGL was superior for GaDOTA vs. F-DOPA. Specificity was comparable between these two imaging modalities and better than for ^18^FDG and ^123^MIBG. The beneficial pharmaceutical properties of ^18^F-DOPA (stable for 12 h, no need for on-site production) and the favorable diagnostic performance suggests ^18^F-DOPA PET/CT as a good alternative to ^68^GaDOTA-based tracers (short half-life, need for on-site generation of 68Ga, not affordable to many hospitals) in metastatic PPGL.

## 1. Introduction

Pheochromocytoma (PCC) and paraganglioma (PGL), together PPGL, are rare catecholamine-secreting tumors originating from chromaffin cells, with a prevalence of 0.2–0.6% in patients with hypertension and about 7% (1.5–14%) in those with adrenal incidentalomas [1]. PPGLs are the most frequently inherited tumors in humans, with up to 40% of patients harboring pathogenic germline variants in 1 of about 20 susceptibility genes [2,3]. Germline genetic testing has been recommended for all patients [4] to allow personalized management [5]. All PPGLs have metastatic potential [6], and, although the prevalence of metastases may be as high as 15–20% depending on the tumor background, metastatic disease has been described in about 8–10% of large non-selected cohorts [3,7]. Approximately 10–15% of patients exhibit metastases at first presentation [3,8], but metastases may occur many years later [3,8]. There are few therapeutic options once metastatic disease has developed [9]. Thus, an early and correct detection of metastases is of paramount importance, especially in patients with genetic disease who may be more prone to metastatic PPGL [5].

Functional imaging, the fusion of positron emission tomography (PET) and computed tomography (CT), in short PET/CT, provide high lesion-to-background resolution and combine functional characteristics with sensitive detection of the tumor. PET/CT with tracers such as [18F]-Fluoro-deoxyglucose (FDG), [18F]-Fluoro-dihydroxyphenylalanine (F-DOPA) or 68Gallium (Ga) radiolabeled 1,4,7,10-tetraazacyclododecane-tetraacetic acid (DOTA)-[Tyrosine^3^, Tyr3] coupled with somatostatin receptor (SSR) agonists octreotide (DOTATOC, edotriotide), DOTA-Tyr3-octreotate (DOTATATE, oxodotreotide) and DOTA-NaI3-octreotide (DOTANOC) have revolutionized diagnosis and follow-up (FU) of PPGL. Functional imaging has become the method of choice for confirming the diagnosis of PPGL, for staging at initial presentation or for restaging and follow-up [10]. These imaging procedures should be considered prior to decision making in patients with metastatic PPGL and in those at high risk of metastatic disease [11] due to a large primary tumor, extra-adrenal PGL, recurrent disease, pathologic germline variants in genes such as the succinate dehydrogenase type B (*SDHB*) or locally advanced disease at first presentation [8]. The type of functional imaging procedure should be tailored to the characteristics of the patient and the tumor, including germline genetic profile, tumor size and location as sensitivities, and specificities vary depending on the context [11,12]. There is debate regarding which tracers are best suited for the diagnosis and/or FU of a specific patient with PPGL. Much of the present knowledge has been gathered through meta-analyses and systematic reviews of small-scale studies, especially in patients with metastatic PPGL [13,14,15].

The aim of this single-center retrospective study was to evaluate the diagnostic accuracy of functional imaging modalities (F-DOPA-, Ga-DOTA based- and FDG-PET/CT) for the diagnosis of metastatic disease in a sample of 57 patients with metastatic PPGL and of 527 patients without PPGL as performed in everyday clinical practice of a large university hospital during long-time FU^123^ Iodine-metaiodobenzylguanidine (MIBG) scintigraphy has been included, given that therapy with ^131^MIBG may be an option in metastatic disease when surgical treatment is no longer an option. In the further course of the text, imaging modalities are named according to the radiolabeled tracer but always refer to fusion imaging with PET/CT.

## 2. Materials and Methods

### 2.1. Participants and Study Design

**Figure 1:**
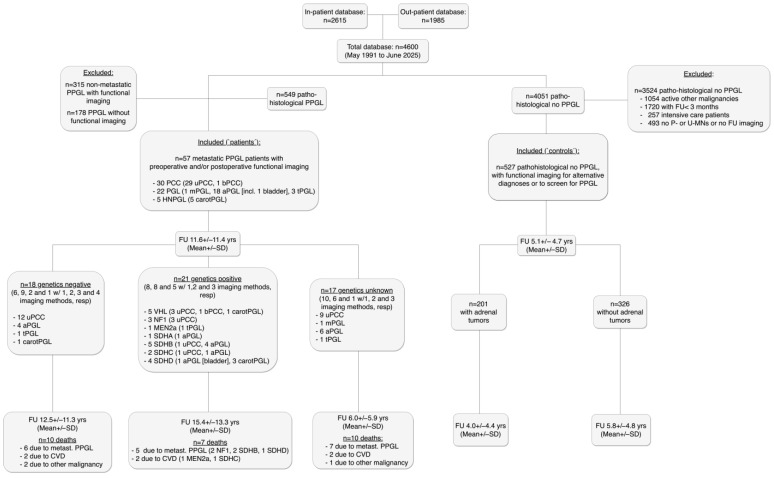
Flow chart of the study cohort. Please note that tumor locations relate to those at first surgery. Abbreviations are those in the main text and as follows: FU—follow-up; SD—standard deviation; uPCC, bPCC—unilateral and bilateral pheochromocytoma; mPGL, aPGL, tPGL—multiple, abdominal and thoracic paraganglioma; carotPGL—carotid paraganglioma; P- or U-MNs—plasma- or 24 h urinary metanephrines; CVD—cardiovascular disease.

All radiological and nuclear medicine findings, surgical reports, out-patient visits and ICD-10 codes, as well as free text including abbreviations relating to PPGL (Supplementary Table S1) of the electronic patient database of the University Hospital Vienna were screened for patients with PPGL and with at least one functional imaging procedure between May 1991 and June 2025 and an FU of at least three months. Survival data as retrieved from the Austrian Death Registry are valid until 31 December 2024. The total database included 4600 patients. A total of 315 of 549 patients with a pathohistological diagnosis of PPGL had to be excluded because there were no metastases, and 178 of these 549 were excluded because there was no functional imaging. A total of 57 (10.4% of all PPGL) patients with metastatic disease (N = 21 at first diagnosis, N = 35 during FU) were included. Mean FU was 11.6 years (Figure 1). Methods of germline genetic analyses of patients with and without PPGL have been published elsewhere [3].

The control group consisted of 527 patients with functional imaging performed for suspicion of PPGL or for other indications, 201 with and 326 without adrenal tumors. Indications to perform functional imaging, best possible methods to exclude PPGL (histopathological proof of non-PPGL in 77 [14.6%], plasma- (P-) and/or 24 h urinary (U-) metanephrines (MNs) within the reference range in 252 [47.8%], no tumor on functional imaging or normal FU imaging in 196 [37.2%] and uneventful FU in 2 patients after 7.3 and 1.8 years, respectively) and main diagnoses of the control group (total and the 201 patients with and 326 without adrenal tumor, respectively) are given in Supplementary Material. Of note, PPGL could be excluded by more than one method in 92.6% of these patients (Supplementary Table S2). Characteristics of the control group are given in Supplementary Tables S2 and S3.

The imaging data were read by two independent nuclear medicine specialists as part of their daily clinical routine incorporating available clinical, biochemical and imaging results (i.e., not blinded to available patient data). The decision on “positive” (metastasis present) or “negative” (metastasis absent) PET/CT results was based on consensus by the endocrine tumor board (consisting of board-certified endocrinologists, endocrine surgeons, nuclear medicine specialists, oncologists, endocrine pathologists, radiologists and radiation oncologists with decades of experience with endocrine tumors) of the University Hospital Vienna (in 48 of the 57 [84%] patients with metastatic PPGL and in 81% of the control patients). For the remainder and for about 60% of MIBG studies, results were used as documented in the electronic patient database. Given that MIBG was the only tracer available in earlier years and based on the high mortality of metastatic PPGL, not every MIBG examination could be followed with PET/CT studies. The adjudication of PET/CT results by tumor board consensus of patients with previous MIBG examinations (in 24 of 59 [40.7%] MIBG studies) included the adjudication of MIBG results as well; however, potential bias comparisons were mitigated due to the mixing of reading paradigms.

Exclusion criteria other than no functional imaging, non-metastatic PPGL and FU < 3 months were treatment for active other malignancy, for active systemic inflammatory disease or intensive care patients (for both the patient and the control groups) and lack of P- or U-MNs or FU imaging when neither histopathology nor biochemical data were available at baseline to establish an alternative diagnosis (control group). No effort was made to assess medication history. Baseline characteristics of the study cohort are given in Table 1 and in Figure 1.

### 2.2. Imaging Protocols

Many generations of SPECT- and PET- imaging hardware with varying protocols were used during the FU period of the present study (1991–2025). Details of the imaging protocols for the PET/CTs with ^18^F-DOPA [16,17,18], with ^18^FDG [19,20] and with ^68^GaDOTA [21] have been previously published. A summary of every tracer is given in Supplementary Material.

### 2.3. Statistics

Categorical variables are presented using number (%) of subjects, continuous data as mean ± standard deviation (SD) or as median (range) depending on data type and distribution. Whenever possible, comparisons were made with the Chi Square test, the Fisher’s exact test, one-way analysis of variance (ANOVA) followed by Tukey ’s multiple comparisons test or the Kruskal–Wallis test followed by Dunn ’s multiple comparisons test, as appropriate.

Calculations of sensitivity and specificity were for all metastases from PPGL together and stratified by metastases in lymph nodes (Lnn), parenchymatous organs and bone, respectively. These calculations are given for the total cohort on the one hand (sensitivity in 57 patients with metastatic PPGL, specificity analyses in these 57 and—given that in PPGL patients the number of non-PPGL lesions were rather low—in 527 patients without PPGL, respectively) as well as divided by germline genetic results (negative, positive, unknown) in patients with metastatic PPGL on the other. Results for each imaging modality (in the case of the GaDOTA-based studies those of GaDOTANOC and GaDOTATOC together) are given based on the number of patients, lesions and imaging procedures. The majority of patients in our study displayed more than one metastatic lesion and had more than one FU imaging procedure (either with the same or another modality). Results of the initial examinations and of all FU imaging studies were analyzed together for all imaging modalities. A lesion was defined false positive either if it was proven to be benign on pathohistological examination (by biopsy or surgery), was not detectable in a succeeding anatomical or functional imaging study not explainable by additional surgery or one of the other non-surgical therapies administered during FU (outlined in Table 1 and Supplementary Figure S1) or if the written report in the patient chart was “overruled” by mutual expert consensus of the specialized tumor board. Lesions were defined false negative if they were progressing in size or number or developed a significant uptake on FU imaging examination and could not be attributed to confounding disease (i.e., other malignancies or NF1). All metastatic lesions were counted for the lesion-based analysis, in the case of bulky Lnn metastases the number of “bulks” when single Lnn could not be delineated any more. Due care was taken to assess the location of the metastases to ensure identical lesions repeatedly assessed during FU were not counted more than once, such that only new lesions were added in the next FU imaging studies.

Fisher’s exact test was used to compare sensitivities and specificities between all four imaging modalities and (separately) between DOPA and GaDOTA-based studies. The 95% confidence intervals (CIs) of sensitivity/specificity were by the hybrid Wilson/Brown method [22]. *p*-values < 0.05 were considered significant. All computations were performed using GraphPad Prism version 10.6.0 for Mac (GraphPad Software, San Diego, CA, USA, www.graphpad.com). Generative artificial intelligence (GenAI) has not been used in this paper.

## 3. Results

### 3.1. Patient Characteristics

Patients with PCC and pathogenic germline variants were younger than those with PGL and negative germline genetic findings (*p* = 0.04) but not compared with the other groups. The total FU duration of patients with HNPGL was shorter than all other groups (*p* < 0.0001). However, FU with imaging was comparable between all patient groups. The number of imaging studies per patient was higher (*p* = 0.003) in those with HNPGL than in all other groups except for PGL with negative germline genetic findings. The total number of imaging studies were higher (*p* = 0.002) for all patients with PCC than for all with PGL. However, when normalized for the number of patients, there was no significant difference. All other baseline characteristics were not different between the patient groups (Table 1).

### 3.2. Therapies During FU (i.e., After First Surgery)

There were 31 (54.4%) patients with PPGL, with a total of 50 additional therapies after first surgery (N = 21 in 16 patients with PCC, N = 18 in 13 with PGL and N = 11 in 2 with HNPGL), either before the first FU imaging study in 5 patients (N = 2 with MIBG, N = 1 with systemic chemotherapy, N = 1 with external radiation and N = 1 with two modalities consisting of additional surgery and selective intraarterial embolization therapy [SIRT]), after (in 26 patients) or both before and after the first FU imaging study (in 3 patients). Nine of these 35 patients had more than one (N = 5 with two, N = 1 with three, N = 2 with four and N = 1 with 7) FU therapy. Details regarding to the temporal relation of all FU therapies to first surgery and to respective preceding imaging studies are given in Table 1 and Supplementary Figure S1a–c.

### 3.3. Diagnostic Performance of Imaging Modalities

A total of 245 imaging studies (Table 1 for details) were evaluated during an FU of up to 29.6 years. The following results refer to patient-, lesion- and imaging-based analyses unless otherwise stated (Table 2 and Table 3).

#### 3.3.1. Patient-Based Analysis

##### For All Metastases Together

Overall sensitivity was 72%, 77%, 75% and 67% for MIBG, F-DOPA, FDG and GaDOTA, respectively. There was no significant difference, either when all modalities were compared or when only F-DOPA and GaDOTA were considered, in the total cohort and divided by location of the primary tumor (Table 2).

Overall specificity was lowest for MIBG (13%) and smaller (*p* = 0.007) than for F-DOPA (78%), FDG (67%) and GaDOTA (100%). Overall specificity in the control group of 527 patients without PPGL was lowest for MIBG (73%). MIBG and FDG (77% specific) performed worse (*p* < 0.0001) than F-DOPA and GaDOTA (both 98% specific). There was no difference in specificity when F-DOPA and GaDOTA were compared to each other (Table 3).

##### For Detection of Metastases in Lnn, Parenchymatous Organs and Bone

There were no significant differences of the performances between the imaging modalities for detection of metastases in Lnn, parenchymatous organs or bone, irrespective of considering all patients or when dividing the cohort by location of the primary tumor or germline genetic results (Supplementary Tables S4 and S6 [sensitivity], Supplementary Tables S5 and S7 [specificity]).

#### 3.3.2. Lesion-Based Analysis

##### For All Metastases Together

Overall sensitivity was 67%, 94%, 98% and 85% for MIBG, F-DOPA, FDG and GaDOTA, respectively. MIBG performed less sensitive (*p* < 0.0001) than the other three modalities to detect all metastases, F-DOPA better (*p* < 0.0001) than GaDOTA in the total cohort (94% vs. 85%), in patients with PCC (96% vs. 74%) but not in patients with PGL or HNPGL (Table 2).

Overall specificity was similar to the patient-based analysis and is shown in Table 3. In summary, there was no difference between F-DOPA and GaDOTA.

##### For Detection of Metastases in Lnn, Parenchymatous Organs and Bone

There was no difference in sensitivity between the imaging modalities for detection of Lnn metastases in the total cohort, in patients with PGL and with HNPGL. However, GaDOTA (74% sensitive) performed worse (*p* = 0.03) than the other three modalities in patients with PCC. Both, MIBG (64%, 74%, 50% and 50% sensitive in the total cohort, in patients with PCC, PGL and HNPGL, respectively) and GaDOTA (67% sensitive in PGL) performed worse (*p* < 0.0001) than the other modalities for the detection of parenchymatous metastases. Similar less sensitive (*p* < 0.0001) results for MIBG (62%, 40% and 13% in the total cohort, in patients with PGL and HNPGL, respectively) and for GaDOTA (21% for patients with PCC) were obtained for the detection of bone metastases (Supplementary Table S4).

Sensitivity of imaging modalities to detect all metastases in patients divided by location of the primary tumor and by germline genetic results are given in Supplementary Table S6. In summary, F-DOPA was as sensitive as GaDOTA in all patient subgroups and performed better (*p* < 0.0001) than GaDOTA in patients with PCC and negative genetic results (95% vs. 71%).

Specificity analyses of imaging modalities for subgroups of patients with PPGL were hampered by the small number of non-metastatic lesions and are given in Supplementary Tables S5 and S7. In summary, specificity of F-DOPA was not different to GaDOTA.

#### 3.3.3. Imaging-Based Analysis

##### For All Metastases Together

Overall sensitivity was 84%, 85%, 93% and 87% for MIBG, F-DOPA, FDG and GaDOTA, respectively. There was no significant difference, either when all modalities were compared or when only F-DOPA and GaDOTA were considered (Table 2).

Overall specificity was similar to the patient-based analysis and is shown in Table 3. In summary, there was no difference between F-DOPA and GaDOTA.

##### For Detection of Metastases in Lnn, Parenchymatous Organs and Bone

Sensitivity to detect Lnn metastases was comparable for all imaging modalities, irrespective of the total cohort or when divided into PCC, PGL and HNPGL. GaDOTA (44% sensitive) performed worse (*p* = 0.004) in PGL and MIBG (50% sensitive) worse (*p* < 0.0001) in HNPGL than the other imaging modalities for detection of parenchymatous metastases. The detection of bone metastases in patients with PGL and HNPGL was worse (*p* = 0.007 and <0.0001) with MIBG (67% and 50% sensitive, respectively), in patients with PCC worse (*p* = 0.02) with GaDOTA and FDG (67% and 60% sensitive, respectively) compared with the other imaging modalities. Sensitivity was not different between F-DOPA and GaDOTA in any patient group, irrespective of metastases in Lnn, parenchymatous organs or bone (Supplementary Table S4).

Sensitivity for detection of all metastases was smaller (*p* = 0.02) for FDG (50%) in patients with PCC and negative genetic results, for MIBG (79%) with PGL and positive genetic results and comparable in the other subgroups (Supplementary Table S6). There was no difference in sensitivity when F-DOPA and GaDOTA were compared to each other in any patient groups.

Specificity analyses of imaging modalities for subgroups of patients with PPGL were hampered by the small number of non-metastatic lesions and are given in Supplementary Tables S5 and S7. In summary, specificity of F-DOPA was not different to GaDOTA.

#### 3.3.4. False Negative and False Positive Results

Details as to false negative and false positive findings are given in Supplementary Table S8.

##### False Negative Results

Four MIBG, one F-DOPA and two GaDOTA studies (in three, one and two patients, respectively) were false positive with respect to a total of 20 local Lnn metastases. Only abdominal Lnn metastases were missed.

Nine MIBG, twelve DOPA, one FDG and seven GaDOTA (in eight, five, one and four patients) were false positive with respect to metastases in parenchymatous organs, MIBG missing 45 lesions, F-DOPA 11, FDG 1 and GaDOTA 11, respectively.

Eight MIBG, five F-DOPA, two FDG and two GaDOTA (in five, two, one and two patients, respectively) missed a total of 145 bone lesions (MIBG 99, F-DOPA 11, FDG 4 and GaDOTA 31). No particular skeletal location was predominant.

##### False Positive Results

False positive results in Lnn were observed with MIBG only (six neurofibromas in three different locations in the neck, the mediastinum and one intervertebral foramen of the spine in one patient with NF1).

Eight MIBG and four F-DOPA studies (in five and three patients, respectively) were false positive in a total of 16 parenchymatous lesions. One lesion each in the thymus, the bowels, the lung, the adrenal and one kidney (the latter first without morphological correlate, a second time leading to the diagnosis of a renal cell carcinoma), respectively, were falsely considered PPGL metastases by MIBG, a total of eleven (four liver, two pancreatic, one renal, two pulmonary and two distant Lnn) lesions by F-DOPA.

There were no false positive results with respect to bone lesions.

## 4. Discussion

To the best of our knowledge, this is the largest study to evaluate the diagnostic performance of functional imaging in patients with metastatic PPGL (N = 57) and in patients in whom PPGL has been excluded (N = 527 controls). F-DOPA-, FDG- and GaDOTA-based PET/CT and MIBG scintigraphy were analyzed over a mean FU of 11.6 years (patients) and 5.1 years (controls), respectively. In the absence of a uniform gold standard, confirmation or exclusion of PPGL in lesions was obtained by best possible means, including pathohistological examination and detailed FU clinical, FU biochemical and FU imaging data for each patient. Sensitivity and specificity were calculated for all patients together as well as divided by germline genetic results and by metastatic sites (Lnn, parenchymatous organs and bone).

GaDOTA and F-DOPA are the two commonly used tracers in patients with PPGL [11]. Thus, the main findings of our study (summarized in Table 4) may be considered the superior sensitivity of F-DOPA vs. GaDOTA for detection of all bone metastases in the total cohort and in patients with PCC, while sensitivity to detect bone metastases in patients with PGL was superior for GaDOTA vs. F-DOPA. Specificity was comparable between these two imaging modalities.

The results of our study are difficult to compare with those obtained by others. First, although there were two GaDOTA-based modalities (GaDOTATOC and GaDOTANOC) performed during the 11.5 years of FU, GaDOTATATE has only recently been available in our hospital and there are no data with that tracer in the present study. GaDOTATATE was introduced in the US (National Institute of Health, NIH) as a research and clinical scan in January 2014 and July 2021, respectively [23] and widely published. Results regarding the performance of the other two GaDOTA tracers are scarce. The diagnostic performance of the three GaDOTA-based tracers are considered comparable [24], which is in line with our findings (comparable performances of GaDOTANOC and -TOC, data given for images studies with both tracers combined). However, discordant results have been observed between GaDOTATATE and GaDOTANOC in a case report [25], which may be related to the former’s approximately nine times higher affinity to SSR2, a receptor mainly overexpressed in PGL [26]. Second, the absence of a gold standard to confirm or exclude PPGL for lesions not pathohistologically investigated (i.e., most metastatic lesions) has led authors to use a composite of all functional and anatomic imaging as an imaging comparator [27,28,29], with obvious implications to internal validity [13]. We could avoid such a design by using comprehensive clinical, biochemical, imaging and pathohistological data during long-term FUs of >11 years to more reliably assess the validity of positive or negative functional imaging results. Third, there is significant variation in how diagnostic performance results are reported in the literature (total cohorts, PCC or PGL, specific pathogenic germline variants, different metastatic sites, patient-based or lesion-based, total number of metastatic lesions or truncated at a particular number) as outlined previously [13]. Our study did not compare imaging modalities head-to-head but aimed to assess their accuracy during everyday clinical practice of comparable real-world conditions. As such, nuclear medicine specialists were not blinded to the results of all preceding imaging studies of a particular patient. This could have led to an overestimation of sensitivity and specificity of FU imaging studies vs. the first. Given that doctors were not blinded to preceding imaging studies with all modalities and given that most patients had multiple functional imaging studies during FU, this may not have confounded our results. The number of preoperative examinations (N = 13 of a total of 245) were too small for a separate analysis. Thus, our data allow for estimates of performance characteristics of imaging modalities for both, the diagnosis of primary metastatic disease and the FU of metastases during long-term FU (with FU imaging studies contributing to almost 95% of the data).

The clinical utility of PET tracers does not only depend on accuracy measures but also on the additional diagnostic information they add to clinical, biochemical, morphological, pathohistological and genetic information already available [13]. We tried to approximate that clinical need by means of a long FU and comprehensive assessment of available data. It has been argued that ruling metastases in or out could be considered clinically more useful than determining the exact number of metastases in patients with widespread metastatic disease [13]. As such, patient- or imaging-based analysis could provide additional information given the categorical outcome (metastatic yes or no) instead of continuous results (number of metastases). Patient-based sensitivity analysis in patients with metastatic PPGL are rarely performed given the low number of patients studied [13]. We were able to provide patient-based analysis. Comparable sensitivities of all imaging modalities were observed by patient-based analysis.

F-DOPA performed equally well to or even better than GaDOTA in our cohort by lesion-based analyses. This is in contrast to some [28,30] but not all [31,32,33] other reports. The sample size in other studies was generally small and ranged from N = 5 patients with metastatic PCC of a cohort of N = 57 with PPGL [31], N = 10 with metastatic PPGL [30], N = 17 with *SDH*x (N = 13) and MEN2a (N = 4)-associated metastatic PPGL [33] to an unknown number of N = 27 patients with recurrent or metastatic PPGL among N = 36 patients with PPGL [32]. The most recent systematic literature review on the diagnostic performance of GaDOTA and F-DOPA focused on studies in different PPGL subtypes comparing PET tracers head-to-head [13]. Eleven studies of consecutive patients with standardized multimodal functional imaging and ten with nonconsecutive patients, individualized imaging or unspecified were included. The number of patients (median, range) with metastatic PPGL was 22 (6–43) and 4 (0–24) of these 11 and 10 studies, respectively. Analyses were mainly lesion-based given the small sample size hindering patient-based evaluation. The limited evidence suggested that GaDOTA was most sensitive for patients with sympathetic metastatic PGL, but it could not be concluded if this also considered non-*SDH*x-related sympathetic metastatic PGL. GaDOTA was considered superior to F-DOPA for metastatic HNPGL and for *SDH*x-associated PPGL in general, respectively. Results were more heterogeneous for metastatic PCC, with some studies reporting F-DOPA to be superior or equal to GaDOTA [13]. The most recent guidelines of the European Association of Nuclear Medicine (EANM) recommend GaDOTATOC/DOTATATE as the first-line tool in patients with metastatic PPGL, F-DOPA as second choice without *SDHB* mutations, FDG as second-line with *SDHB* pathogenic variants and MIBG to examine whether patients are eligible for MIBG therapy [11].

Our study has strengths and limitations. Our cohort consisted of a large number of patients undergoing functional imaging for a broad range of reasons, and patients were diagnosed and followed for >11 years (patients) and >5 years (controls). Previously, functional imaging was primarily used for selected patients with sympathetic PGL, HNPGL or a small cross-sectional cohort of metastatic PPGL, which may have skewed the literature towards these patient groups even though considered a minority [13]. Second, the addition of more than 500 patients without PPGL allowed to evaluate reliable specificity data of functional imaging for the diagnosis and exclusion of PPGL for the first time. Comparable specificities for F-DOPA vs. GaDOTA were observed and for both were better than MIBG and FDG. Third, given that pathohistological confirmation is often unfeasible and unethical for metastatic PPGL, imaging results were compared with best possible surrogates (i.e., a combination of pathohistological, biochemical, imaging and clinical data during FU) rather than comparing PET tracers to each other or to a composite of all functional and anatomic imaging, threatening internal validity of reported data [13]. There are limitations of our study: First, the performance of PET scanners has improved considerably since their introduction to clinical practice, limiting conclusions as to diagnostic accuracy over time. However, this improvement should apply to all PET tracers and may have caused little bias in our study given that the proportional use of both F-DOPA- and GaDOTA-based imaging has increased in parallel until present. Still, the lack of head-to-head comparisons and the combining of preoperative and FU imaging in our study may have affected internal validity. Second, the number of F-DOPA studies (101) was almost double that of GaDOTA (58) in our PPGL cohort, which may have resulted in skewed sensitivity results (either at the expense or in favor of GaDOTA). Third, about 60% of MIBG results relied on the written reports documented in the patient charts, while PET/CT results were adjudicated by tumor board consensus in 84% of patients with PPGL, which may have biased comparisons. The adjudication of PET/CT results by tumor board consensus of patients with previous MIBG examinations (in 24 of 59 [40.7%] MIBG studies) included the adjudication of MIBG results as well, to mitigate potential bias comparisons due to the mixing of reading paradigms. Given that MIBG was the only tracer available in earlier years and based on the high mortality of metastatic PPGL, not every MIBG examination could be followed with PET/CT studies. Fourth, combining preoperative and FU imaging was considered appropriate given the small number of preoperative (N = 13 of all 245) examinations but may have biased performance characteristics, which—in addition to the fact that results were not blinded to preceding images—may have led to an overestimation of sensitivity and specificity of individual imaging modalities. However, comparisons may not have been compromised much given that preoperative and FU imaging studies were analyzed together for all imaging modalities and based on preoperative examinations contributing to only 5.3% of imaging data. It should be stressed, though, that our data allowed to estimate performance characteristics of imaging modalities for the combined diagnosis of primary metastatic disease and metastases developing during long-term FU only. Last, we could not analyze diagnostic performance for subgroups other than those with germline genetic results and location of metastatic sites. This is an inherent problem given the low prevalence of metastatic PPGL. In addition, given the known genotype–phenotype and possible modality–phenotype relationships in PPGL, genetic and tumor type heterogeneity may have been significant confounders.

## 5. Conclusions

The main findings of our study (summarized in Table 4) may be considered the superior sensitivity of F-DOPA vs. GaDOTA for the detection of all and bone metastases in the total cohort and in patients with PCC, while sensitivity to detect bone metastases in patients with PGL was superior for GaDOTA vs. F-DOPA. Specificity was comparable between these two imaging modalities, and superior to MIBG and FDG. Given the beneficial pharmaceutical properties of F-DOPA (stable for 12 h, no need for on-site production) and the favorable diagnostic performance, F-DOPA PET/CT may be more readily available and a good alternative to GaDOTA-based tracers (short half-life, need for on-site generation of ^68^Ga, not affordable to many hospitals) for functional imaging of metastatic PPGL

## Figures and Tables

**Table 1:** Characteristics of 57 patients with PPGL and metastatic disease.

	PCC, Genet Neg (N = 11)	PCC, Genet Pos (N = 9)	PCC, Genet Unknown (N = 10)	PGL, Genet Neg (N = 5)	PGL, Genet Pos (N = 9)	PGL, Genet Unknown (N = 8)	HNPGL, Genet Pos (N = 5)	*p*-Value
Age at 1st surgery, years (mean ± SD)	48.8 ± 13.8	24.7 ± 12.4	36.7 ± 13.0	50.8 ± 11.6	35.0 ± 21.2	46.7 ± 17.0	28.5 ± 10.6	0.04
Male sex (%)	2 (18.2)	5 (55.6)	6 (60.0)	2 (40.0)	5 (55.6)	5 (62.5)	3 (60.0)	0.10 *
Tumor size (cm), mean ± SD (range)	7.1 ± 3.2 (3.5–13.5)	11.0 ± 4.3 (5.8–17.0)	10.3 ± 4.5 (5.5–18.0)	8.3 ± 2.9 (4.5–12)	5.5 ± 3.1 (2.0–10.0)	7.6 ± 6.6 (1.5–18)	4.4 ± 1.1 (3.5–6.0)	0.21
Metastatic disease at 1st surgery, N (%)	3 (27.3)	3 (33.3)	4 (40.0)	4 (80.0)	3 (33.3)	4 (50.0)	0	0.72 *
Location of primary metastatic disease (N)	Loc Lnn (1), Loc Lnn + lungs (1), dissem. (1)	Loc Lnn (2), Loc Lnn +liver (1)	Loc Lnn (2), Liver (1), Tu-thr. into RA + lungs (1)	Loc Lnn (2), Liver (1), Liver + distant Lnn (1)	Loc Lnn (2), Loc Lnn + bone (1)	Loc muscle (1), Loc muscle + bone (1), dissem. (2)		
N pathogenic variants: *VHL*, *NF1*, *RET*, *SDHA*, *SDHB*, *SDHC*, *SDHD*, *CHEK2*, resp.	n.a.	4, 3, 0, 0, 1, 1, 0, 0	n.a.	n.a.	0, 0, 1, 1, 5, 1, 0, 1	n.a.	2, 0, 0, 0,0, 0, 3, 0	-
FU duration, total (years) mean ± SD (range)	14.4 ± 11.2 (1.3–35.4)	15.5 ± 13.2 (0.4–32.7)	7.7 ± 7.1 (1.2–21.2)	7.8 ± 7.3 (2.0–19.6)	7.7. ± 7.7 (1.7–26.9)	3.6 ± 3.3 (0.7–9.1)	31.0 ± 8.8 (17.8–41.1)	<0.0001 **
FU with imaging (years), mean ± SD (range)	2.7 ± 1.9 (0.7–6.4)	6.7 ± 9.7 (0.4–29.6)	1.8 ± 1.7 (0.6–5.9)	7.6 ± 8.8 (1.9–17.7)	4.5. ± 4.2 (0.3–10.4)	1.1 ± 1.5 (0.3–3.7)	6.9 ± 4.6 (1.6–11.7)	0.2
Ther after 1st OP, N (∆)	N = 9 (6pts)	N = 5 (4pts)	N = 7 (6pts)	N = 5 (3pts	N = 7 (4pts)	N = 6 (6pts)	N = 11 (2pts)	
- Additional surgery	1(∆ +1), 1^#^(∆+ 2)	1^#^(∆+1), 2(∆+2), 1^#^(∆+3)		2^#^(∆+1), 1^#^(∆+3)	2^#^(∆+2), 1(∆+3), 1^#^(∆+4)	2(∆+1), 1(∆+3)	1^#^(∆—1)	
- ^131^MIBG	1(∆—1), 2(∆+2), 1(∆+3), 1^#^(∆+4)	1(∆+2)	3(∆+1), 1(∆+2), 1^#^(∆+3)		1^#^(∆+3)	1(∆+1)		
- ^177^Lu-DOTA	1^#^(∆+7)			1^#^(∆+8)		1(∆+1)	1^#^(∆+1), 1^#^(∆+4,+12)	
- External radiation	1^#^(∆+2)			1^#^(∆+2)	1^#^(∆+8)	1(+4)	1^#^(∆—1,+1), 1^#^(∆+1,+11)	
- Chemotherapy			1^#^(∆—1), 1(∆+2)		1^#^(∆+5)		1^#^(∆+1),	
- SIRT							1^#^(∆—1,+20)	
Overall death, N (%)	5 (45.5)	2 (22.2)	6 (60.0)	4 (80.0)	4 (44.4)	4 (50.0)	2 (40.0)	0.69 *
Death of metast PPGL, N (%)	3 (27.3)	2 (22.2)	5 (50.0)	3 (60.0)	2 (22.2)	3 (37.5)	1 (20.0)	0.99 *
PreOP imaging, N pts (method)	1 (1 DOPA)	3 (1 MIBG, 2 DOPA)	3 (3 MIBG)	2 (1 FDG, 1 GaOTA)	3 (2 MIBG, 1 DOPA)	0	1 (1 MIBG)	0.85 *
FU imaging, N pts (%)	11 (100.0)	9 (100.0)	10 (100.0)	4 (80.0)	9 (100.0)	8 (100.0)	5 (100.0)	0.85 *
FU imaging studies, N	46	43	20	17	39	17	63	0.003 **
N FU imaging studies per pt, mean ± SD (range)	4.6 ± 2.3 (2–9)	4.8 ± 3.9 (1–13)	2.2 ± 0.9 (1–4)	3.4 ± 3.1 (0–7)	6.6 ± 5.7 (1–19)	2.1 ± 1.4 (1–5)	13.2 ± 10.8 (2–24)	0.0003 ***
-MIBG, N/in N pts (range per pt)	12/7 (0–3)	14/4 (0–11)	13/9 (0–3)	4/3 (0–2)	6/4 (0–3)	7/4 (0–3)	3/3 (0–1)	
-DOPA, N/in N pts (range per pt)	25/9 (0–8)	28/8 (0–8)	3/2 (0–2)	9/4 (0–5)	5/4 (0–2)	6/5 (0–2)	25/5 (0–20)	
-FDG, N/in N pts (range per pt)	3/2 (0–2)	0	2/2 (0–1)	1/1	16/1 (0–16)	2/1 (0–2)	3/2 (0–2)	
-GaDOTA, N/in N pts (range per pt)	6/4 (0–2)	1/1	2/2 (0–1)	3/1 (0–3)	12/5 (0–4)	2/1 (0–2)	32/3 (0–21)	

Abbreviations are those used in the text and as follows: genet neg, pos—genetics negative, positive, N—number, pt—patient, Loc Lnn—local lymph nodes, dissem.—disseminated metastatic disease (i.e., in local Lnn, in parenchymatous organs and in bone), Tu-thr.—tumor-thrombus, VCI—inferior vena cava, RA—right atrium, resp—respectively, *VHL*—von-Hippel–Lindau, *NF1*—neurofibromatosis type 1, *RET*—rearranged during transfection protocooncogene, *SDH,-C,-D*—succinate dehydrogenase type A to D, *CHEK2*—checkpoint kinase 2, pts—patients, n.a.—not applicable, Ther—therapy, metast — metastatic, ^177^Lu—Lutetium-177, SIRT—selective intraarterial radioembolization therapy. The symbol ∆ refers to the temporal relationship of therapy to first surgery (∆—1 prior to first surgery) or after 1st, 2nd, 3rd imaging during FU (∆+1, ∆+2, ∆+3,…), respectively. * by Chi square test, ** by Fisher’s exact test, comparing all PCC vs. all PGL, *** comparisons to HNPGL only (*p* > 0.05 for comparisons between non-HNPGL), ^#^ patients with more than one FU therapy modality.

**Table 2:** Sensitivity (95% confidence intervals of sensitivity) of imaging modalities to detect all metastases (i.e., in lymph node, parenchymatous organs and bone together) by patient-, lesion- and imaging-based analyses in 57 patients with metastatic PPGL and divided by location of the primary tumor at first surgery.

	MIBG	F-DOPA	FDG	GaDOTA	*p* Value	*p* Value *
All (N = 57)						
Patient-based	0.72 (0.56–0.84)	0.77 (0.63–0.87)	0.75 (0.47–0.91)	0.67 (0.47–0.82)	0.81	0.34
Lesion-based	0.67 (0.63–0.71)	0.94 (0.92–0.96)	0.98 (0.95–0.99)	0.85 (0.81–0.88)	<0.0001	<0.0001
Imaging-based	0.84 (0.76–0.89)	0.85 (0.80–0.90)	0.93 (0.84–0.97)	0.87 (0.80–0.92)	0.33	0.64
PCC (N = 30)						
Patient-based	0.81 (0.60–0.92)	0.76 (0.55–0.89)	0.75 (0.30–0.99)	0.60 (0.31–0.83)	0.66	0.35
Lesion-based	0.92 (0.87–0.95)	0.96 (0.92–0.98)	0.87 (0.71–0.95)	0.74 (0.67–0.80)	<0.0001	<0.0001
Imaging-based	0.90 (0.81–0.95)	0.79 (0.70–0.86)	0.78 (0.45–0.96)	0.75 (0.53–0.89)	0.20	0.69
PGL (N = 22)						
Patient-based	0.64 (0.39–0.84)	0.72 (0.49–0.88)	1.0 (0.51–1.0)	0.7 (0.40–0.89)	0.58	0.90
Lesion-based	0.47 (0.40–0.56)	0.87 (0.80–0.92)	1.0 (0.98–1.0)	0.92 (0.86–0.95)	<0.0001	0.29
Imaging-based	0.76 (0.61–0.87)	0.87 (0.73–0.94)	1.0 (0.92–1.0)	0.79 (0.62–0.90)	0.007	0.41
HNPGL (N = 5)						
Patient-based	0.50 (0–09-0.91)	1.0 (0.57–1.0)	0.5 (0.09–0.91)	0.75 (0.30–0.99)	0.29	0.24
Lesion-based	0.24 (0.14–0.39)	1.0 (0.96–1.0)	0.98 (0.91–1.0)	0.98 (0.92–1.0)	<0.0001	0.24
Imaging-based	0.50 (0.19–0.81)	1.0 (0.93–1.0)	0.6 (0.23–0.93)	0.95 (0.86–0.99)	<0.0001	0.10

Abbreviations are those in the text. Please note that all patients with HNPGL harbored pathogenic germline variants. * for comparison of DOPA vs. GaDOTA.

**Table 3:** Specificity (95% confidence intervals of specificity) of imaging modalities to exclude all metastases (i.e., in lymph node, parenchymatous organs and bone together) by patient-, lesion- and imaging-based analyses in 57 patients with metastatic PPGL, divided by location of the primary tumor at first surgery and in 527 control (CON) patients without PPGL.

	MIBG	F-DOPA	FDG	GaDOTA	*p* Value	*p* Value *
All (N = 57)						
Patient-based	0.13 (0.01–0.47)	0.78 (0.55–0.91)	0.67 (0.12–0.98)	1.0 (0.44–1.0)	0.007	0.36
Lesion-based	0.11 (0.02–0.31)	0.90 (0.81–0.95)	0.42 (0.23–0.64)	1.0 (0.61–1.0)	<0.0001	0.42
Imaging-based	0.07 (0.01–0.32)	0.86 (0.76–0.92)	0.50 (0.09–0.91)	1.0 (0.85–1.0)	<0.0001	0.06
PCC (N = 30)						
Patient-based	0 (0.0–0.49)	0.78 (0.45–0.89)	1.0 (0.05–1.0)	1.0 (0.05–1.0)	0.04	0.60
Lesion-based	0 (0.0–0.23)	0.85 (0.72–0.93)	1.0 (0.65–1.0)	1.0 (0.18–1.0)	<0.0001	0.68
Imaging-based	0 (0.0–0.44)	0.76 (0.61–0.87)	1.0 (0.05–1.0)	1.0 (0.05–1.0)	0.002	0.65
PGL (N = 22)						
Patient-based	0 (0.0–0.56)	0.19 (0.08–0.38)	0 (0.0–0.95)	n.a.	n.a.	n.a.
Lesion-based	0 (0.0–0.49)	0.93 (0.79–0.99)	0 (0.0–0.26)	n.a.	n.a.	n.a.
Imaging-based	0 (0.0–0.32)	0.89 (0.67–0.98)	0 (0.0–0.82)	n.a.	n.a.	n.a.
HNPGL (N = 5)						
Patient-based	1.0 (0.05–1.0)	1.0 (0.05–1.0)	1.0 (0.05–1.0)	1.0 (0.18–1.0)	n.a.	n.a.
Lesion-based	1.0 (0.18–1.0)	1.0 (0.18–1.0)	1.0 (0.05–1.0)	1.0 (0.51–1.0)	n.a.	n.a.
Imaging-based	1.0 (0.44–1.0)	1.0 (0.85–1.0)	1.0 (0.05–1.0)	1.0 (0.94–1.0)	n.a.	n.a.
CON, all (N = 527)						
Patient-based	0.73 (0.57–0.85)	0.98 (0.95–0.99)	0.77 (0.70–0.82)	0.98 (0.95–1.0)	<0.0001	0.74
Lesion-based	0.71 (0.56–0.83)	0.96 (0.93–0.98)	0.75 (0.69–0.81)	0.98 (0.95–1.0)	<0.0001	0.25
Imaging-based	0.73 (0.57–0.85)	0.97 (0.94–0.98)	0.82 (0.77–0.86)	0.99 (0.97–0.99)	<0.0001	0.09
CON, adr. tu. (N = 201)						
Patient-based	0.28 (0.13–0.51)	0.93 (0.85–0.97)	0.65 (0.55–0.73)	0.90 (0.74–0.96)	<0.0001	0.68
Lesion-based	0.70 (0.48–0.86)	0.91 (0.83–0.95)	0.65 (0.57–0.73)	0.92 (0.79–0.97)	<0.0001	0.99
Imaging-based	0.72 (0.49–0.88)	0.92 (0.85–0.96)	0.68 (0.59–0.75)	0.90 (0.82–0.95)	<0.0001	0.81
CON, no adr. tu. (N = 326)						
Patient-based	0.74 (0.51–0.88)	0.99 (0.97–1.0)	0.97 (0.89–0.99)	1.0 (0.98–1.0)	<0.0001	0.99
Lesion-based	0.74 (0.51–0.88)	0.99 (0.97–1.0)	0.97 (0.89–0.99)	1.0 (0.98–1.0)	<0.0001	0.99
Imaging-based	0.74 (0.51–0.88)	1.0 (0.97–1.0)	0.98 (0.94–1.0)	1.0 (0.99–1.0)	<0.0001	0.28

Abbreviations are those in the text. Please note that all patients with HNPGL harbored pathogenic germline variants. Abbreviations: n.a. – not applicable, * for comparison of DOPA vs. GaDOTA.

**Table 4:** Summary of performance comparison F-DOPA vs. GaDOTA (all patients, all metastases).

	Sensitivity, Lesion-Based		
	F-DOPA	GaDOTA	*p*-Value
all pts, all meta	0.94	0.85	<0.0001
all PCC, all meta	0.96	0.74	<0.0001
all pts, bone meta	0.97	0.79	<0.0001
all PCC, bone meta	1.0	0.21	<0.0001
all PGL, bone meta	0.86	1.0	0.001

## Data Availability

Data will be provided in the Supplementary Material.

## Supplementary Materials

The following supporting information can be downloaded at: https://www.mdpi.com/article/10.3390/cancers17233855/s1, Table S1: Terms for the search query of the electronic patient database of the University Hospital Vienna; Table S2: Best possible methods to exclude PPGL, indications to imaging and main diagnoses in 527 control patients (total as well as divided into 201 with and 326 without adrenal tumors); Table S3. Characteristics of 527 patients without PPGL; Table S4. Sensitivity (95% confidence intervals of sensitivity) of imaging modalities to detect lymph node (Lnn), parenchymatous and bone metastases obtained by patient-, lesion- and imaging-based analyses in 57 patients with metastatic PPGL and divided by location of the primary tumor at first surgery; Table S5. Specificity (95% confidence intervals of specificity) of imaging modalities to exclude metastases in local lymph nodes (Lnn), in parenchymatous organs (par) and in bone obtained by patient-, lesion- and imaging-based analyses in 57 patients with metastatic PPGL and divided by location of the primary tumor at first surgery; Table S6. Sensitivity (95% confidence intervals of sensitivity) of imaging modalities to detect all metastases obtained by patient-, lesion- and imaging-based analyses in 30 patients with metastatic PCC and 22 with metastatic PGL divided by germline genetic results; Table S7. Specificity (95% confidence intervals of specificity) of imaging modalities to exclude all metastases obtained by patient-, lesion- and imaging-based analyses in 30 patients with metastatic PCC and 22 with metastatic PGL divided by germline genetic results; Table S8. Details of false positive and false negative imaging results; Figure S1. Details and temporal relationship of imaging studies of 30 patients with metastatic PCC (Supplementary Figure S1a), of 22 with metastatic PGL (Supplementary Figure S1b) and of 5 with metastatic HNPGL (Supplementary Figure S1c) divided by germline genetic results.
