# Supplementary material for: Metastatic Pheochromocytoma/Paraganglioma: Diagnostic Performance of Functional Imaging (18F-Fluoro-L-DOPA-, 68Ga-DOTA- and 18F-Fluoro-Deoxyglucose-Based PET/CT) and of 123I-MIBG Scintigraphy in 57 Patients and 527 Controls During Long-Term Follow-Up
Source: Cancers (Basel). 2025 Nov 30;17(23):3855. doi: 10.3390/cancers17233855 (PMC12691327; doi:10.3390/cancers17233855)
Supplement: Supplementary file 1 [file cancers-17-03855-s001.zip › cancers-4010820-supplementary.pdf]

Supplementary Material to Scheuba A et al.:

Metastatic pheochromocytoma/paraganglioma: diagnosis by functional imaging (^{18}F-Fluoro-L-DOPA-,^{68}Ga-DOTA- and ^{18}F-Fluoro-Deoxyglucose-Based PET/CT) and byMIBG in 57 patients and 527 controls during long-term follow-up

Supplementary Text

1. Patient selection process

The electronic database of the University Hospital Vienna (a primary, secondary and tertiary referral center serving a population of approximately 2.5 million in eastern Austria) between May 1991 and June 2025 were screened for ICD-codes (www.icd10cmtool.cdc.gov) as well as for free text including abbreviations related to PPGL (Supplementary Table S1). By means of 26 ICD-10 codes as well as 37, 42 and 31 terms, words and abbreviations (Supplementary Table S1) there were 22130, 227875, 188351 and 212781 entries identified of the total electronic hospital database (ICD-10 codes), of discharge letters of in-patients, of discharge reports of out-patients and of radiological reports, respectively. After removal of duplicates, of patients with no relation to PPGL, without functional imaging or with FU<3 months, the hospital charts of 4600 patients (2615 in-patients, 1985 out-patients) were studied including >300 patients with PPGL reported previously (Ref. 3 and 8 of the original manuscript) which have been followed prospectively by one of us (W.R.) over the past approximately 10 years. The flowchart of our database is given in Figure 1 of the original manuscript.

2. Functional imaging

Functional imaging modalities of this study were performed as part of everyday routine clinical care of patients, in accordance with the established protocols and standard operating procedures of the Department of Biomedical-Imaging and Image-guided Therapy of our University Hospital. Many generations of SPECT- and PET-scanners with varying protocols were used during the FU period of the present study (1991-2025). Details of the imaging protocols for the PET/CTs with ^{18}F-DOPA (refs. 16-18 of the main mansucript), with ^{18}FDG (refs. 19, 20 of the main manuscript) and with ^{68}GaDOTA (ref. 21 of the main manuscript) have been published previously.

Image reconstruction was performed in accordance with the in-clinic standard operating procedures of the respective generations of scanners. The presence of tracer accumulation in

non-physiological sites was identified as a positive finding. The utilisation of SUV values was exclusively confined to the domain of FDG imaging. The predominantly used protocols are summarized here:

The ^{123}I-mIBG scan was performed ca. 24h after i.v. injection of 200 MBq ^{123}I-mIBG and after pretreatment with potassium iodide on a Siemens Intevo SPECT/CT scanner. The ^{18}F-DOPA PET/CT was performed 60 min after i.v. injection of 200 MBq ^{18}F-DOPA on a Siemens Biograph True Point Scanner Scanner or a Siemens Biograph Vision 600 or Siemens Biograph Vision Quadra scanner. For this scan patients were in a fasting condition. SSTR-ligand PET/CT scanning was performed 60 minutes after the i.v. administration of 200 MBq ^{68}Ga-DOTA-NOC or ^{68}Ga-DOTA-TOC on a Siemens Biograph True Point Scanner or a Siemens Biograph Vision 600 or Siemens Biograph Vision Quadra scanner. For this scan patients were in a fasting condition.^{18}F-FDG PET/CT imaging was performed after the i.v. injection of ~~2,5-33~~ 2.5–3.5 MBq/kg (bodyweight) of ^{18}F-FDG. The blood sugar levels were under 130 mg/dl. For this scan patients were in a fasting condition for at least 6h.

The PET/CT scans were interpreted by nuclear medicine physicians and radiology physicians with experience in this field not blinded to the clinical data. Due to the fact that the patients were undergoing routine scanning for staging purposes, it was not possible to blind the image interpretation to the relevant clinical data.

3. Methods to best possible exclusion of PPGL, indications for functional imaging and main diagnoses of 527 control patients

Patients of the control group were assigned free of PPGL by histopathology (77, 14.6%), plasma- (P-) and/or 24h urinary (U-) metanephrines (MNs) within the normal reference range (252, 47.8%), normal FU imaging results (196, 37.2%) and an uneventful FU (two patients, 0.4%). 159 of 252 (63.1%) patients with normal biochemical findings also displayed normal FU imaging results (Supplementary Table S2). Of note, PPGL could be excluded in 488 (92.6%) of control patients either by more than one method or repeatedly by normal biochemical and/or normal FU imaging results. Imaging results were assigned free of PPGL if there was no lesion or there was no history of PPGL, the patient displayed P- and/or U-MNs within the normal reference range, was free of symptoms or with normal FU imaging results.

Indications to perform functional imaging were different between the 201 patients with and 326 without adrenal tumors. Suspicious adrenal, retroperitoneal and intrathoracic tumors together accounted for 37.3% of reasons to perform functional imaging in patients with adrenal

tumors and none in those without. The latter were screened more often for malignancy (26.1 vs. 8.5%, p<0.0001), for metastases during FU of previous non-PPGL malignancies (44.2 vs. 10.9%, p<0.0001) or for PPGL because of adrenergic symptoms (9.8% vs. 2.5%, p=0.001), the former more often for suspected PPGL based on abnormal P-MNs and/or U-MNs (16.9 vs. 6.1%, p=0.0001) and because of hypertension including hypertensive crises (23.9 vs. 13.8%, p=0.005).

Main diagnoses of patients without adrenal tumors included more non-PPGL malignancies (54.3 vs. 14.5%, p<0.0001), more other diagnoses (22.4 vs. 6.0%, p<0.0001) and more hypertensive crises (16.4 vs. 10.1%, p=0.04) but less adrenal pathologies (none vs. 51.9%) than in those with adrenal tumors. The percentage of patients with hereditary syndromes were comparable between the two groups of control patients (p=0.16). Characteristics of control subjects are given in Supplementary Tables S2 and S3.

4. Therapies during FU (i.e. after first surgery)

There were 31 (54.4%) PPGL patients with a total of 50 additional therapies after first surgery (n=21 in 16 patients with PCC, n=18 in 13 with PGL and n=11 in 2 with HNPGL), either before the first FU imaging study in 5 patients (n=2 with ^{131}MIBG, n=1 with systemic chemotherapy, n=1 with external radiation and n=1 with two modalities consisting of additional surgery and selective intraarterial embolization therapy [SIRT]), after (in 26 patients) or both, before and after the first FU imaging study (in 3 patients). Nine of these 35 patients had more than one (n=5 with two, n=1 with three, n=2 with four and n=1 with eight) FU therapies.

Among the 5 patients with two therapies were two with two FU operations, one with systemic chemotherapy followed by [131I]-MIBG (^{131}MIBG) therapy, one with external radiation followed by [177]Lutetium (^{177}Lu)DOTATAE therapy and one with systemic chemotherapy followed by external radiation. One patient had three FU therapies con-sisting of surgery followed by ^{131}MIBG therapy and a third surgical procedure, two pa-tients four FU therapies: surgery followed by external radiation, ^{131}MIBG- and ^{177}LuDOTATAE therapy in one and external radiation followed by ^{177}LuDOTATATE therapy, another external radiation and systemic chemotherapy in another patient. One patient had 7 FU therapies consisting of additional surgery and SIRT (before first FU imaging), external radiation, ^{177}LuDOTATATE, another external radiation, another ^{177}LuDOTATATE therapy and another SIRT (after first FU imaging).

Details as to the temporal relation of all FU therapies to first surgery and to respective preceding imaging studies are given in Table 1 of the main text and in Supplementary Figures S1a-S1c.

Supplementary Tables and Figures.

Supplementary Table S1. Terms for the search query of the electronic patient database of the University Hospital Vienna.

Supplementary Table S2. Best possible methods to exclude PPGL, indications to imaging and main diagnoses in 527 control patients (total as well as divided into 201 with and 326 without adrenal tumors).

Supplementary Table S3. Characteristics of 527 patients without PPGL.

Supplementary Table S4. Sensitivity (95% confidence intervals of sensitivity) of imaging modalities to detect lymph node (Lnn), parenchymatous and bone metastases obtained by patient-, lesion- and imaging based analyses in 57 patients with metastatic PPGL and divided by location of the primary tumor at first surgery.

Supplementary Table S5. Specificity (95% confidence intervals of specificity) of imaging modalities to exclude metastases in local lymph nodes (Lnn), in parenchymatous organs (par) and in bone obtained by patient-, lesion- and imaging based analyses in 57 patients with metastatic PPGL and divided by location of the primary tumor at first surgery.

Supplementary Table S6. Sensitivity (95% confidence intervals of sensitivity) of imaging modalities to detect all metastases obtained by patient-, lesion- and imaging based analyses in 30 patients with metastatic PCC and 22 with metastatic PGL divided by germline genetic results.

Supplementary Table S7. Specificity (95% confidence intervals of specificity) of imaging modalities to exclude all metastases obtained by patient-, lesion- and imaging based analyses in 30 patients with metastatic PCC and 22 with metastatic PGL divided by germline genetic results.

Supplementary Table S8. Details of false positive and false negative imaging results.

Supplementary Figure S1. Details and temporal relationship of imaging studies of 30 patients with metastatic PCC (Supplementary Figure S1a), of 22 with metastatic PGL (Supplementary Figure S1b) and of 5 with metastatic HNPGL (Supplementary Figure S1c) divided by germline genetic results.

ICD-10 code<br><br>(total electronic database)	coded disease	Free text incl abbreviations (discharge letters, in-patients)	Free text incl abbreviations (discharge reports, out-patients)	Free text incl abbreviations (radiological reports)
C71.1	Bösartige NB - Frontallappen	CHEK2	CHEK2	DLST
C74.1	Bösartige NB - Nebennierenmark	DLST	Chemodectom	EPAS
C75.4	Bösartige Neubildung: Glomus caroticum	DNMT3A	Chemodektom	FH
C75.5	Bös.NB - Glomus aorticum und sonst. Paraganglien	EPAS	DLST	gangliom
D35.0	Gutartige Neubildung: Nebenniere	FH	DNMT3A	Glomus
D35.0B	Z.n. Adrenalektomie unilateral	gangliom	EGLN	Glomus caroticum
D35.0C	Z.n. Adrenalektomie bilateral	Glomus	EPAS	Glomus vagale
D35.5	Gutartige NB - Glomus caroticum	Glomus aorticum	FH	Glomustumor
D44.1	NB unsich.od.unbek.Verh. - Nebenniere	Glomus caroticum	gangliom	MAX
D44.6	Glomus caroticum dext.	Glomus vagale	Glomus	MEN
D44.7	NB unsich.od.unbek.Verh.Glomus aorticum	Glomustumor	Glomus aorticum	Multiple endokrine Neoplasie
D44.8	Neubildung unsicheren oder unbekannten Verhaltens: Beteiligung mehrerer endokriner Drüsen	KIF1B	Glomus caroticum	Neurofibrom
D44.8A	Multiple Endokrine Neoplasie	MAX	Glomus vagale	Neurofibromatose
D44.8B	MEN 1	MEN	Glomustumor	NF 1
D44.8C	MEN 2	Multiple endokrine Neoplasie	KIF1B<br>MAX	NF1
E27.5	Nebennierenmarküberfunktion, Hypersekretion von...	Neurofibrom		Paragangliom

I15.2	Hypertonie als Folge von endokrinen Krkh.	Neurofibromatose	MDH2	Phäo
I15.2A	Phäochromozytom	NF 1	MEN	Phäochromocytom
I15.2B	familiäres Phäochromozytom	NF1	Multiple endokrine	Phäochromozytom
	(VHL, PGL, MEN 2, Neurofibromatose)		Neoplasie	
I15.2C	Succinat-Dehydrogenase-Mutation	Paragangliom	Neurofibrom	Pheo
I15.2D	MEN 2	Phäo	Neurofibromatose	Pheochromozytom
Q85.0	Neurofibromatose (nicht bösartig), von-Reckling...	Phäochromocytom	NF 1	Recklinghausen
Q85.0A	Neurofibromatose	Phäochromozytom	NF1	RET
Q85.8	sonst. Phakomatosen, anderenorts nicht klassifi...	Pheo	Paragangliom	RET-Protoonkogen
Q85.8A	Von Hippel Lindau	Pheochromozytom	Phäo	SDHA
Q85.8B	Sturge-Weber-Syndrom	Recklinghausen	Phäochromocytom	SDHB
		RET	Phäochromozytom	SDHC
		RET-Protoonkogen	Pheo	SDHD
		SDHA, SDHAF2	Pheochromozytom	VHL
			Recklinghausen	von Hippel
		SDHB	RET	von-Hippe
		SDHC	RET-Protoonkogen	
		SDHD	SDHA, SDHAF2	
		TMEM127	SDHB, SDHC	
		VHL	SDHD, SLC25A11	
		von Hippel	TMEM127, VHL	
		von-Hippe	von Hippel	
			von-Hippe	

Supplementary Table S1. Terms for the search query of the electronic patient database of the University Hospital Vienna.

CON (n=527)		
**Best possible exclusion of PPGL**	**Indication to imaging**	**Main diagnosis**
Normal patho-histology: 77 (14.6%)	Suspicious adrenal tumor: 64 (12.1)	Malign. adrenal tumors: 18 (3.4%)
Normal biochemistry: 252 (47.8%)	Suspicious retroperitoneal tumor: 10 (1.9%)	9 ACC
(159 also w/ normal FU imagings)	Suspicious intrathoracic tumor: 1 (0.2%)	5 lymphomas
Normal FU imagings: 196 (37.2%)	Screening for malignancy: 102 (19.4%)	3 leiomyosarcomas
Uneventful FU: 2 (0.4%), after	9 in ectopic Cushing	1 melanoma
7.3yrs, 1.8 yrs	12 in NF1	Benign adrenal tumors: 71 (13.5%)
	19 in MEN1	40 adrenal adenomas, non-funct.
	19 in carriers of pathogenic variants	6 Conn´ adenomas
	(2 SDHB, 3 SDHC, 2 SDHD, 5 RET	3 Cushing´ adenomas
	4 VHL, 2 CHEK2, 1 MAX)	6 ganglioneuromas
	7 in systemic vasculitis	3 schwannomas
	2 in deep vein thrombosis	5 myelolipomas
	2 in polyneuropathy	3 pseudocysts
	6 in fever of unknown origin	3 Castleman tumors
	4 in nodular goiter (screening for MTC)	1 sarcoidosis
	1 Cowden syndrome	1 normal adrenal
	4 suspicious head-and-neck tumors	Adrenal metastases of other malignancies: 15 (2.9%)
	(3 jugular, 1 tympanal)	(8 NSCLC, 3 breast ca., 1 RCC, 1 HCC,
	17 suspected NET	2 nasopharyngeal carcinoma)

FU of previous other malignancy: 166 (31.5%)
70 abdominal NET, 7 pulm. NET,
75 MTC, 3 Lymphoma
2 NF1 with malignant nerve sheet tumors
2 renal cell carcinoma
5 multiple myeloma
1 struma ovarii
1 GIST

Abnormal biochemistry; 54 (10.3%)
(19 P-MNs, 30 U-MNs, 5 U-CATs)

Arterial hypertension: 93 (17.6%)
(62 w/ crisis, 31 new)

Symptoms: 37 (7.0%)
(12 w/ abnormal biochem.,
4 w/ hypertensive crisis,
3 w/ systemic vasculitis,
13 w/ anxiety disorder)

Previous other malignancies: 206 (39.1%)
(100 abdominal NET, 87 MTC, 9 pulm. NET, 3 lymphomas, 2 RCC, 2 MM, 1 GIST,
1 colonic carc., 1 prostate carc.)

Arterial hypertension: 70 (13.3%)
(66 w/ crisis, most w/ type-2 diabetes, CHD, aortic. dissection, chronic renal insuff., renal art. stenosis and/or other CVD)

Hereditary syndromes: 62 (11.7%)
(4 *VHL*, 16 *NF1*, 4 *SDHB*, 2 *SDHC*, 1 *SDHD*,
18 MEN1, 14 MEN2, 1 *MAX*, 2 *CHEK2*)

Other: 85 (16.1%)
(13 anxiety disord., 9 chronic renal insuff.,
7 Cushing, 7 orthost. dysregul., 8 systemic vasculitis, 4 dementia, 3 heredit. angioedema,
3 DTC, 3 lymphadenompathy, 3 vertigo, 3 AF,
3 cholesteatoma, 2 vasospasm, 2 PHP, 2 FUO,
2 Carney complex, 2 pancreatitis, 2 meningeoma,
2 PNP, 2 LVD, 1 FU/bariatr surgery, 1 ALS
1 endolymphatic sac tumor)

CON w/ adrenal tu (n=201)		
**Best possible exclusion of PPGL**	**Indication to imaging**	**Main diagnosis**
Normal patho-histology: 64 (31.8%)	Suspicious adrenal tumor: 64 (31.8%)	Malign. adrenal tumors: 18 (9.0%)
Normal biochemistry: 116 (57.7%)	Suspicious retroperitoneal tumor: 10 (5.0%)	9 ACC
(28 also w/ normal FU imagings)	Suspicious intrathoracic tumor: 1 (0.5%)	5 lymphomas
Normal FU imagings: 19 (9.5%)	Screening for malignancy: 17 (8.5%)	3 leiomyosarcomas
Uneventful FU: 2 (1.0%), after 7.3yrs, 1.8 yrs	4 in ectopic Cushing	1 melanoma
	2 in NF1	Benign adrenal tumors: 71 (35.4%)
	6 in MEN1	40 adrenal adenomas, non-funct.
	2 in systemic vasculitis	6 Conn´ adenomas
	2 in deep vein thrombosis	3 Cushing´ adenomas
	1 in fever of unknown origin)	6 ganglioneuromas
	FU of previous other malignancy: 22 (10.9%)	3 schwannomas
	8 abdominal NET	5 myelolipomas
	5 MTC	3 pseudocysts
	3 Lymphoma	3 Castleman tumors
	2 NF1 with malignant nerve sheet tumors	1 sarcoidosis
	2 renal cell carcinoma	1 normal adrenal
	2 multiple myeloma	Adrenal metastases of other malignancies: 15 (7.5%)
	Abnormal biochemistry; 34 (16.9%)	(8 NSCLC, 3 breast ca., 1 RCC, 1 HCC,

	(8 P-MNs, 21 U-MNs, 5 U-CATs)	2 nasopharyngeal carcinoma)
	Arterial hypertension: 48 (23.9%)	Previous other malignancies: 29 (14.5%)
	(28 w/ crisis, 20 new)	(13 MTC, 11 abdominal NET, 2 RCC,
	Symptoms: 5 (2.5%)	2 multiple myelomas, 1 GIST)
		Arterial hypertension: 37 (18.4%)
		(33 w/ crisis, 4 new w/ type-2 diabetes)
		Hereditary syndromes: 19 (9.5%)
		(6 *NF1*, 6 MEN1, 7 MEN2a)
		Other: 12 (6.0%)
		(4 anxiety disorder, 4 dementia, 2 systemic
		vasculitis, 1 FU/bariatric surgery, 1 ALS)
	**CON w/o adrenal tu (n=326)**	
**Best possible exclusion of PPGL**	**Indication to imaging**	**Main diagnosis**
Normal patho-histology: 13 (4.0%)	Screening for malignancy: 85 (26.1%)	Hereditary syndromes: 43 (13.2%)
Normal biochemistry: 136 (41.7%)	5 in ectopic Cushing	(4 *VHL*, 10 *NF1*, 4 *SDHB*, 2 *SDHC*, 1 *SDHD*,
(131 also w/ normal FU imagings)	10 in NF1	12 MEN1, 7 MEN2 [carrier], 1 *MAX*, 2 CHEK2)
Normal imagings: 177 (54.3%)	13 in MEN1	Previous other malignancies: 177 (54.3%)
	19 in carriers of pathogenic variants	(89 abdom. NET, 9 pulmon. NET, 74 MTC,
	(2 SDHB, 3 SDHC, 2 SDHD, 5 RET	3 lymphoma, 1 colonic carc., 1 prostate carc.,
	4 VHL, 2 CHEK2, 1 MAX)	Hypertensive crisis: 33 (10.1%)
	5 in systemic vasculitis	(all w/ type-2 diabetes, chron. renal insuff., CHD,

2 in polyneuropathy

5 in fever of unknown origin)

4 in nodular goiter (screening for MTC)

1 Cowden syndrome

4 suspicious head-and-neck tumors

(3 jugular, 1 tympanal)

17 suspected NET

FU of previous other malignancy: 144 (44.2%)

62 abdominal NET

7 pulmonary NET

70 MTC

3 multiple myeloma

1 struma ovarii

Abnormal biochemistry: 20 (6.1%)

(11 P-MNs, 9 U-MNs)

Arterial hypertension: 45 (13.8%)

(34 w/ crisis, 11 new)

Symptoms: 32 (9.8%)

(12 w/ abnormal biochem.,

4 w/ hypert crisis, 3 w/ systemic vasculitis,

13 w/ anxiety disorder)

renal artery stenosis, aortic diss. and/or other CVD)

Other: 73 (22.4%)

(9 anxiety disord., 9 chronic renal insuff., 7 Cushing,

7 orthost. dysregul., 6 systemic vasculitis, 3 DTC,

3 AF, 3 heredit. angioedema, 3 lymphadenompathy,

3 vertigo, 3 cholesteatoma, 2 PHP, 2 LVD,

2 vasospasm, 2 FUO, 2 Carney complex,

2 pancreatitis, 2 meningeoma,

2 polyneuropathy, 1 endolymphatic sac tumor)

0

Supplementary Table S2. Best possible methods to exclude PPGL, indications to imaging and main diagnoses in 527 control patients (total as well as divided into 201 with and 326 without adrenal tumors). Abbreviations: w/=with, w/o=without, FU=follow-up, *NF1*=neurofibromatosis type 1, MEN1=multiple endocrine neoplasia type 1, MEN2a=multiple endocrine neoplasia type 2a, *SDHB, -C, -D*=succinate dehydrogenase-deficiency type B, C, D, *RET*=rearranged-during-transfection proto-oncogene, *VHL*=von Hippel-Lindau, *CHEK2*=checkpoint kinase 2, *MAX*=myc-associated factor X (also called myc proto-oncogene), MTC=medullary thyroid carcinoma, NET=neuroendocrine tumor, GIST=gastro-intestinal stromal tumor, P-MNs=plasma metanephrines, U-MNs=24-hour urinary metanephrines, U-CATs=24-hour urinary catecholamines, ACC=adrenocortical carcinoma, NSCLC=non small-cell lung cancer, RCC=renal cell carcinoma, HCC=hepato-cellular carcinoma, MM=multiple myeloma, CHD=coronary heart disease, CVD=cardiovascular disease, DTC=differentiate thyroid carcinoma, AF=atrial fibrillation, PHP=primary hyperparathyroidism, FUO=fever of unknown origin, PNP=polyneuropathy, LVD=left ventricular dysfunction, ALS=amyotrophic lateral sclerosis.

	**CON all (n=527)**	**CON with adr.tu. (n=201)**	**CON without adr.tu (n=326)**	**p-value**
Male sex, n (%)	242 (45.9)	91 (45.3)	151 (46.3)	0.85
FU duration	5.1±4.7	4.0±4.4	5.8±4.8	0.64
Tumor size, cm	3.9±3.1	3.9±3.1	n.a.	n.a.

Supplementary Table S3. Characteristics of 527 patients without PPGL. Abbreviations: CON=control group, adr.tu.=adrenal tumor, n.a.= not applicable.

	MIBG Lnn	DOPA Lnn	FDG Lnn	GaDOTA Lnn	p value	p value*	MIBG Par	DOPA Par	FDG Par	GaDOTA Par	p value	p value*	MIBG Bone	DOPA Bone	FDG Bone	GaDOTA Bone	p value	p value*
**All**	**(N=57)**																	
Patient	0.86	0.85	0.83	0.69	0.63	0.40	0.79	0.80	0.80	0.71	0.93	0.70	0.75	0.91	0.89	0.82	0.51	0.59
based	(0.65-0.95	(0.67-0.94)	(0.44-0.99)	(0.42-0.87)			(0.60-0.91)	(0.61-0.91)	(0.38-0.99)	(0.45-0.88)			(0.55-0.88)	(0.72-0.98	(0.57-0.99)	(0.52-0.97)		
Lesion	0.94	0.92	0.98	0.85	0.11	0.14	0.64	0.89	1.0	0.90	<0.0001	0.83	0.62	0.97	0.98	0.79	<0.0001	<0.0001
based	(0.85-0.98	(0.84-0.96)	(0.87-1.0)	(0.75-0.91)			(0.55-0.72)	(0.81-0.94)	(0.88-1.0)	(0.84-0.94)			(0.56-0.68)	(0.94-0.98	(0.95-0.99)	(0.73-0.85)		
Imaging	0.90	0.83	0.94	0.74	0.30	0.36	0.81	0.82	0.94	0.84	0.62	0.99	0.83	0.93	0.93	0.96	0.11	0.70
based	(0.74-0.96	(0.70-0.91)	(0.72-1.0)	(0.54-0.88)			(0..66-0.90)	(0.72-0.89)	(0.73-1.0)	(0.70-0.93)			(0.70-0.91)	(0.84-0.97	(0.77-0.99)	(0.86-0.99)		
**PCC all**	**(N=30)**																	
Patient	0.86	0.77	1.0	0.67	0.68	0.99	0.81	0.83	1.0	0.80	0.93	0.99	1.0	0.90	0.67	0.5	0.07	0.18
based	(0.60-0.98	(0.50-0.92)	(0.18-1.0)	(0.30-0.94)			(0.57-0.93)	(0.55-0.97)	(0.18-1.0)	(0.38-0.99)			(0.74-1.0)	(0.60-1.0)	(0.12-0.98)	(0.09-0.91)		
Lesion	0.96	0.88	1.0	0.74	0.03	0.15	0.74	0.91	1.0	0.97	<0.0001	0.14	1.0	1.0	0.82	0.21	<0.0001	<0.0001
based	(0.85-0.99	(0.76-0.94)	(0.51-1-0)	(0.57-0.85)			(0.63-0.83)	(0.80-0.96)	(0.57-1.0)	(0.91-0.99)			(0.97-1.0)	(0.97-1.0)	(0.62-0.93)	(0.12.-0.36)		
Imaging	0..91	0.77	1.0	0.71	0.47	0.99	0.80	0.74	1.0	0.86	0.73	0.68	1.0	0.87	0.60	0.67	0.02	0.23
based	(0.71-0.98	(0.59-0.88)	(0.18-1.0)	(0.36-0.95)			(0.63-0.91)	(0.58-0.85)	(0.18-1.0)	(0.47-0.99)			(0.88-1.0)	(0.72-0.95	(0.23-0.93)	(0.30-0.94)		
**PGL all**	**(N=22)**																	
Patient	0.88	0.90	1.0	0.88	0.85	0.99	0.83	0.73	1.0	0.5	0.46	0.60	0.56	0.86	1.0	1.0	0.15	0.99
based	(0.49-0.99	(0.60-0.99)	(0.44-1.0)	(0.38-0.99)			(0.44-0.99)	(0.43-0.90)	(0.18-1.0)	(0.19-0.81)			(0.27-0.81)	(0.49-0.99	(0.51-1-0)	(0.51-1-0)		
Lesion	0.89	1.0	1.0	0.94	0.12	0.44	0.50	0.78	1.0	0.67	0.004	0.39	0.40	0.86	1.0	1.0	<0.0001	0.001
based	(0.67-0.98	(0.85-1.0)	(0.90-1.0)	(0.73-1.0)			(0.34-0.66)	(0.58-0.90)	(0.80-1.0)	(0.50-0.80)			(0.32-0.49)	(0.76-0.93	(0.97-1.0)	(0.95-1-0)		
Imaging	0.88	0.93	1.0	0.91	0.68	0.99	0.89	0.75	1.0	0.44	0.01	0.20	0.67	0.91	1.0	1.0	0.007	0.99
based	(0.53-0.99	(0.70-1.0)	(0.77-1.0)	(0.62-1.0)			(0.57-0.99)	(0.47-0.91)	(0.79-1.0)	(0.19-0.73)			(0.45-0.83)	(0.62-1.0)	(0.84-1.0)	(0.70-1.0)		
**HNPGL**	**(N=5)**																	
Patient	n.a.	1.0	0	0.5	0.15	0.40	0.50	1.0	0	1.0	0.15	0.99	0.5	1.0	1.0	1.0	0.12	0.99
based		(0.44-1.0)	(0.0-0.95)	(0.03-0.97)			(0.03-0.97)	(0.18-1.0)	(0.0-0.95)	(0.44-1.0)			(0.09-0.91)	(0.57-1.0)	(0.93-1.0)	(0.44-1.0)		
Lesion	n.a.	1.0	1.0	0.5	0.10	0.33	0.50	1.0	0.88	1.0	0.0006	0.99	0.13	1.0	1.0	1.0	<0.0001	0.99
based		(0.51-1-0)	(0.0-0.95)	(0.03-0.97)			(0.03-0.97)	(0.78-1.0)	(0.53-0.99)	(0.79-1.0)			(0.05-0.29)	(0.95-1.0)	(0.18-1.0)	(0.92-1.0)		
Imaging	n.a.	1.0	0	0.4	0.13	0.20	0.50	1.0	0	1.0	<0.0001	0.99	0.5	1.0	1.0	1.0	<0.0001	0.99
based		(0.44-1.0)	(0.0-0.95)	(0.07-0.77)			(0.03-0.97)	(0.86-1.0)	(0.0-0.95)	(0.85-1.0)			(0.09-0.91)	(0.87-1.0)	(0.44-1.0)	(0.90-1.0)		

Supplementary Table S4. Sensitivity (95% confidence intervals of sensitivity) of imaging modalities to detect lymph node (Lnn), parenchymatous and bone metastases obtained by patient-, lesion- and imaging based analyses in 57 patients with metastatic PPGL and divided by location of the primary tumor at first surgery. Abbreviations are those in the text and as follows: Lnn, lymph nodes; par, parenchymatous; n.a., not applicable. Please note that all HNPGL patients harbored pathogenic germline variants. *comparisons of ^{18}F-DOPA vs. ^{68}GaDOTA.

	MIBG Lnn	DOPA Lnn	FDG Lnn	GaDOTA Lnn	p value	p value*	MIBG Par	DOPA Par	FDG Par	GaDOTA Par	p value	p value*	MIBG Bone	DOPA Bone	FDG Bone	GaDOTA Bone	p value	p Value*
**All**	**(N=57)**																	
Patient based	0.17<br>(0.01-0.56)	0.50<br>(0.09-0.91)	0<br>(0.0-0.95	1.0<br>(0.05-1.0)	0.29	0.99	0<br>(0.0-0.49)	0.88<br>(0.64-0.98)	0.67<br>(0.12-0.98)	1.0<br>(0.18-1.0)	0.07	0.99	n.a.	1.0<br>(0.05-1.0)	n.a.	n.a.	n.a.	n.a.
Lesion based	0.29<br>(0.06-0.64)	0.86<br>(0.47-0.99)	0<br>(0.0-0.32	1.0<br>(0.18-1.0)	0.002	0.99	0<br>(0.0-0.24)	0.91<br>(0.81-0.96)	0.73<br>(0.43-0.90)	1.0<br>(0.51-1.0)	<0.0001	0.99	n.a.	n.a.	n.a.	n.a.	n.a.	n.a.
Imaging based	0.13<br>(0.01-0.47)	0.60<br>(0.23-0.93)	0<br>(0.0-0.95	1.0<br>(0.74-1.0)	0.001	0.08	0<br>(0.0-0.39)	0.87<br>(0.77-0.93)	0.67<br>(0.12-0.98)	1.0<br>(0.74-1.0)	<0.0001	0.35	n.a.	1.0<br>(0.18-1.0)	n.a.	n.a.	n.a.	n.a.
**PCC**	**(N=30)**																	
Patient based	1.0<br>(0.18-1.0)	1.0<br>(0.05-1.0)	1.0<br>(0.18-1.0	n.a.	0.08	n.a.	0<br>(0.0-0.56)	0.78<br>(0.45-0.96)	1.0<br>(0.05-1.0)	1.0<br>(0.05-1.0)	0.07	0.99	n.a.	1.0<br>(0.05-1.0)	n.a.	n.a.	n.a.	n.a.
Lesion based	1.0<br>(0.18-1.0)	1.0<br>(0.51-1.0)	1.0<br>(0.51-1-0	n.a.	0.01	n.a.	0<br>(0.0-0.26)	0.84<br>(0.69-0.92)	1.0<br>(0.65-1.0)	1.0<br>(0.18-1.0)	<0.0001	0.99	n.a.	n.a.	n.a.	n.a.	n.a.	n.a.
Imaging based	1.0<br>(0.18-1.0)	1.0<br>(0.18-1.0)	1.0<br>(0.18-1.0	n.a.	0.99	n.a.	0<br>(0.0-0.56)	0.74<br>(0.57-0.85)	1.0<br>(0.05-1.0)	1.0<br>(0.05-1.0)	0.047	0.99	n.a.	1.0<br>(0.18-1.0)	n.a.	n.a.	n.a.	n.a.
**PGL**	**(N=22)**																	
Patient based	0<br>(0.0-0.56)	0.33<br>(0.02-0.88)	0<br>(0.0-0.95	n.a.	0.46	n.a.	0<br>(0.0-0.95)	1.0<br>(0.57-1.0)	0<br>(0.0-0.95)	n.a.	0.03	n.a.	n.a.	n.a.	n.a.	n.a.	n.a.	n.a.
Lesion based	0<br>(0.0-0.56)	0.67<br>(0.12-0.98)	0<br>(0.0-0.32	n.a.	0.01	n.a.	0<br>(0.0-0.95)	1.0<br>(0.87-1.0)	0<br>(0.0-0.56)	n.a.	<0.0001	n.a.	n.a.	n.a.	n.a.	n.a.	n.a.	n.a.
Imaging based	0<br>(0.0-0.44)	0.33<br>(0.02-0.88)	0<br>(0.0-0.95	n.a.	0.32	n.a.	0<br>(0.0-0.56)	1.0<br>(0.80-1.0)	0<br>(0.0-0.95)	n.a.	<0.0001	n.a.	n.a.	n.a.	n.a.	n.a.	n.a.	n.a.
**HNPGL**	**(N=5)**																	
Patient based	1.0<br>(0.05-1.0)	n.a.	n.a.	1.0<br>(0.05-1.0)	0.99	n.a.	n.a.	1.0<br>(0.18-1.0)	1.0<br>(0.05-1.0)	1.0<br>(0.05-1.0)	0.99	0.99	n.a.	n.a.	n.a.	n.a.	n.a.	n.a.
Lesion based	1.0<br>(0.18-1.0)	n.a.	n.a.	1.0<br>(0.18-1.0)	0.99	n.a.	n.a.	1.0<br>(0.05-1.0)	1.0<br>(0.05-1.0)	1.0<br>(0.18-1.0)	0.99	0.99	n.a.	n.a.	n.a.	n.a.	n.a.	n.a.
Imaging based	1.0<br>(0.05-1.0)	n.a.	n.a.	1.0<br>(0.74-1.0)	0.99	n.a.	n.a.	1.0<br>(0.05-1.0)	1.0<br>(0.05-1.0)	1.0<br>(0.72-1.0)	0.99	0.99	n.a.	n.a.	n.a.	n.a.	n.a.	n.a.

Supplementary Table S5. Specificity (95% confidence intervals of specificity) of imaging modalities to exclude metastases in local lymph nodes (Lnn), in parenchymatous organs (par) and in bone obtained by patient-, lesion- and imaging based analyses in 57 patients with metastatic PPGL and divided by location of the primary tumor at first surgery. Abbreviations are those in the text and as follows: Lnn, lymph nodes; par, parenchymatous; n.a., not applicable. Please note that all HNPGL patients harbored pathogenic germline variants. *comparisons of ^{18}F-DOPA vs. ^{68}GaDOTA.

	MIBG	DOPA	FDG	GaDOTA	p value	p value*
**PCC, genet. neg.**	**(N=11)**					
Patient based	1.0 (0.65-1.0)	0.73 (0.43-0.90)	0.50 (0.03-0.97)	0.60 (0.23-0.93)	0.29	0.61
Lesion based	1.0 (0.96-1.0)	0.95 (0.91-0.98)	0.20 (0.01-0.63)	0.71 (0.63-0.78)	<0.0001	<0.0001
Imaging based	1.0 (0.87-1.0)	0.86 (0.75-0.93)	0.50 (0.09-0.91)	0.75 (0.47-0.91)	0.02	0.35
**PCC, genet. pos.**	**(N=9)**					
Patient based	0.50 (0.09-0.91)	0.75 (0.41-0.96)	n.a.	1.0 (0.05-1.0)	0.53	0.57
Lesion based	0.87 (0.72-0.94)	0.95 (0.88-0.98)	n.a.	1.0 (0.18-1.0)	0.24	0.74
Imaging based	0.88 (0.69-0.96)	0.67 (0.51-0.79)	n.a.	1.0 (0.05-1.0)	0.15	0.48
**PCC, unknown genet.**	**(N=10)**					
Patient based	0.80 (0.49-0.97)	1.0 (0.18-1.0)	1.0 (0.18-1.0)	0.50 (0.09-0.91)	0.40	0.22
Lesion based	0.85 (0.76-0.91)	1.0 (0.83-1.0)	1.0 (0.87-1.0)	0.92 (0.74-0.99)	0.046	0.20
Imaging based	0.83 (0.66-0.92)	1.0 (0.51-1.0)	1.0 (0.57-1.0)	0.71 (0.36-0.95)	0.45	0.24
**PGL, genet. neg.**	**(N=5)**					
Patient based	0.50 (0.03-0.97)	0.60 (0.23-0.93)	1.0 (0.05-1.0)	0.50 (0.03-0.97)	0.84	0.81
Lesion based	0.60 (0.23-0.93)	0.57 (0.39-0.73)	1.0 (0.74-1.0)	0.89 (0.77-0.95)	0.004	0.54
Imaging based	0.67 (0.12-0.98)	0.67 (0.42-0.85)	1.0 (0.18-1.0)	0.50 (0.19-0.81)	0.37	0.14
**PGL, genet. pos.**	**(N=9)**					
Patient based	0.63 (0.31-0.86)	0.83 (0.44-0.99)	1.0 (0.18-1.0)	0.71 (0.36-0.95)	0.67	0.61
Lesion based	0.45 (0.34-0.57)	0.87 (0.76-0.93)	1.0 (0.96-1.0)	0.91 (0.82-0.96)	<0.0001	0.31
Imaging based	0.79 (0.52-0.92)	0.93 (0.70-1.0)	1.0 (0.91-1.0)	0.85 (0.64-0.95)	0.02	0.44
**PGL, unknown genet.**	**(N=8)**					
Patient based	0.75 (0.30-0.99)	0.71 (0.36-0.95)	1.0 (0.05-11.0)	1.0 (0.05-1.0)	0.85	0.51
Lesion based	0.48 (0.39-0.58)	0.85 (0.67-0.94)	1.0 (0.94-1.0)	1.0 (0.83-1.0)	<0.0001	0.07
Imaging based	0.79 (0.52-0.92)	0.82 (0.52-0.97)	1.0 (0.61-1.0)	1.0 (0.44-1.0)	0.54	0.43

Supplementary Table S6. Sensitivity (95% confidence intervals of sensitivity) of imaging modalities to detect all metastases obtained by patient-, lesion- and imaging based analyses in 30 patients with metastatic PCC and 22 with metastatic PGL divided by germline genetic results. Abbreviations are those in the text and as follows: genet. neg., germline genetics negative; genet. pos., germline genetics postive; n.a., not applicable. *comparisons of ^{18}F-DOPA vs. ^{68}GaDOTA.

	MIBG	DOPA	FDG	GaDOTA	p value	p value*
**PCC, genet. neg.**	**(N=11)**					
Patient based	n.a.	1.0 (0.68-1.0)	1.0 (0.05-1.0)	n.a.	0.22	n.a.
Lesion based	n.a.	1.0 (0.05-1.0)	1.0 (0.65-1.0)	n.a.	0.99	n.a.
Imaging based	n.a.	1.0 (0.51-1.0)	1.0 (0.05-1.0)	n.a.	0.99	n.a.
**PCC, genet. pos.**	**(N=9)**					
Patient based	0 (0.0-0.56)	0.75 (0.41-0.96)	n.a.	1.0 (0.05-1.0)	0.06	0.57
Lesion based	0 (0.0-0.24)	0.85 (0.72-0.93)	n.a.	1.0 (0.18-1.0)	<0.0001	0.56
Imaging based	0 (0.0-0.49)	0.74 (0.57-0.85)	n.a.	1.0 (0.05-1.0)	0.01	0.55
**PCC, unknown genet.**	**(N=10)**					
Patient based	n.a.	n.a.	n.a.	n.a.	n.a.	n.a.
Lesion based	n.a.	n.a.	n.a.	n.a.	n.a.	n.a.
Imaging based	n.a.	n.a.	n.a.	n.a.	n.a.	n.a.
**PGL, genet. neg.**	**(N=8)**					
Patient based	0 (0.0-0.95)	0.5 (0.09-0.91)	n.a.	n.a.	0.36	n.a.
Lesion based	0 (0.0-0.95)	0.5 (0.03-0.97))	n.a.	n.a.	0.008	n.a.
Imaging based	0 (0.0-0.82)	0.5 (0.09-0.91)	n.a.	n.a.	0.07	n.a.
**PGL, genet. pos.**	**(N=9)**					
Patient based	0 (0.0-0.82)	1.0 (0.18-1.0)	1. 0 (0.05-1.0)	n.a.	0.08	n.a.
Lesion based	0 (0.0-0.56)	1.0 (0.65-1.0)	0 (0.0-0.26)	n.a.	<0.0001	n.a.
Imaging based	0 (0.0-0.56)	1.0 (0.79-1.0)	0 (0.0-0.82)	n.a.	0.0002	n.a.
**PGL, unknown genet.**	**(N=8)**					
Patient based	n.a.	1.0 (0.05-1.0)	n.a.	n.a.	n.a.	n.a.
Lesion based	n.a.	1.0 (0.68-1.0)	n.a.	n.a.	n.a.	n.a.
Imaging based	n.a.	1.0 (0.18-1.0)	n.a.	n.a.	n.a.	n.a.

Supplementary Table S7. Specificity (95% confidence intervals of specificity) of imaging modalities to exclude all metastases obtained by patient-, lesion- and imaging based analyses in 30 patients with metastatic PCC and 22 with metastatic PGL divided by germline genetic results. Abbreviations are those in the text and as follows: genet. neg., germline genetics negative; genet. pos., germline genetics postive; n.a., not applicable. *comparisons of ^{18}F-DOPA vs. ^{68}GaDOTA.

**Lnn**<br>false positive			**Lnn**<br>false negative		
Modality, N (in N pts)	N lesions per imaging study	Location, N lesions	Modality, N (in N pts)	N lesions per imaging study	Location, N lesions
1 M	6	2 jugular NF	1 M	1	1 abd Lnn
		2 mediastinal NF	1 M (same pt)	1	1 abd Lnn
		2 For intervert thor 9/10	1 M	2	2 abd Lnn
			1 M	2	2 abd Lnn
			1 D	5	5 abd Lnn
			1 G	8	8 abd Lnn
			1 G	1	1 abd Lnn
**Parenchymatous**<br>false positive			**Parenchymatous**<br>false negative		
Modality, N (in N pts)	N lesions per imaging study	Location, N lesions	Modality, N (in N pts)	N lesions per imaging study	Location, N lesions
1 M	1	1 bowel	1 M	1	1 adr ipsilat
2 M (1pt)	1	1 pulmonary	1 M	4	4 liver
2 M (1 pt)	1	1 adrenal	1 M	1	1 adr contralat
2 M (1pt)	1	1 kidney (1st time no morphol corr, later RCC)	2 M (1pt)	1	1 VCI thrombus
1 M	1	1 thymus	1 M	2	1 pulmonary
1 D	2	2 distant Lnn			1 distant Lnn
1 D	2	2 pancreas	1 M	12	12 distant Lnn
1 D (same pt)	1	1 kidney (RCC)	1 M	7	7 pulmonary
1 D	6	2 pulmonary	1 M	17	17 pulmonary
		4 liver	1 D	3	3 pulmonary
			1 D	1	1 liver
			2 D (1pt)	2	2 pulmonary
			7 D (1pt)	3	1 VCI wall
					2 liver
			1 D	2	1 liver
					1 distant Lnn
			1 F (same pt)	1	1 distant Lnn
			2 G (1pt)	2	2 liver
			3 G (1pt)	1	1 distant Lnn
			1 G	3	2 pulmonary
					1 diaphragma
			1 G (same pt as with 17 pulm)	5	5 pulmonary
**Bone**<br>false positive			**Bone**<br>false negative		
Modality, N (in N pts)	N lesions per imaging study		Modality, N (in N pts)	N lesions per imaging study	
none	none		1 M	8	
			2 M (1pt)	12	
			3 M (1pt)	52	
			1 M	25	
			1 M	2	
			1 D (same pt as with 8 lesions)	9	
			4 D (1pt)	2	
			2 F (1pt)	4	
			1 G	1	
			1 G	30	

Supplementary Table S8. Details of false positive and false negative imaging results. Abbreviations are those in the text and as follows: N=number, M=^{131}MIBG; D=^{18}F-DOPA, F=^{18}FDG, G=^{68}GaDOTA, pt=patient, pulm= pulmonary, VCI= inferior vena cava.

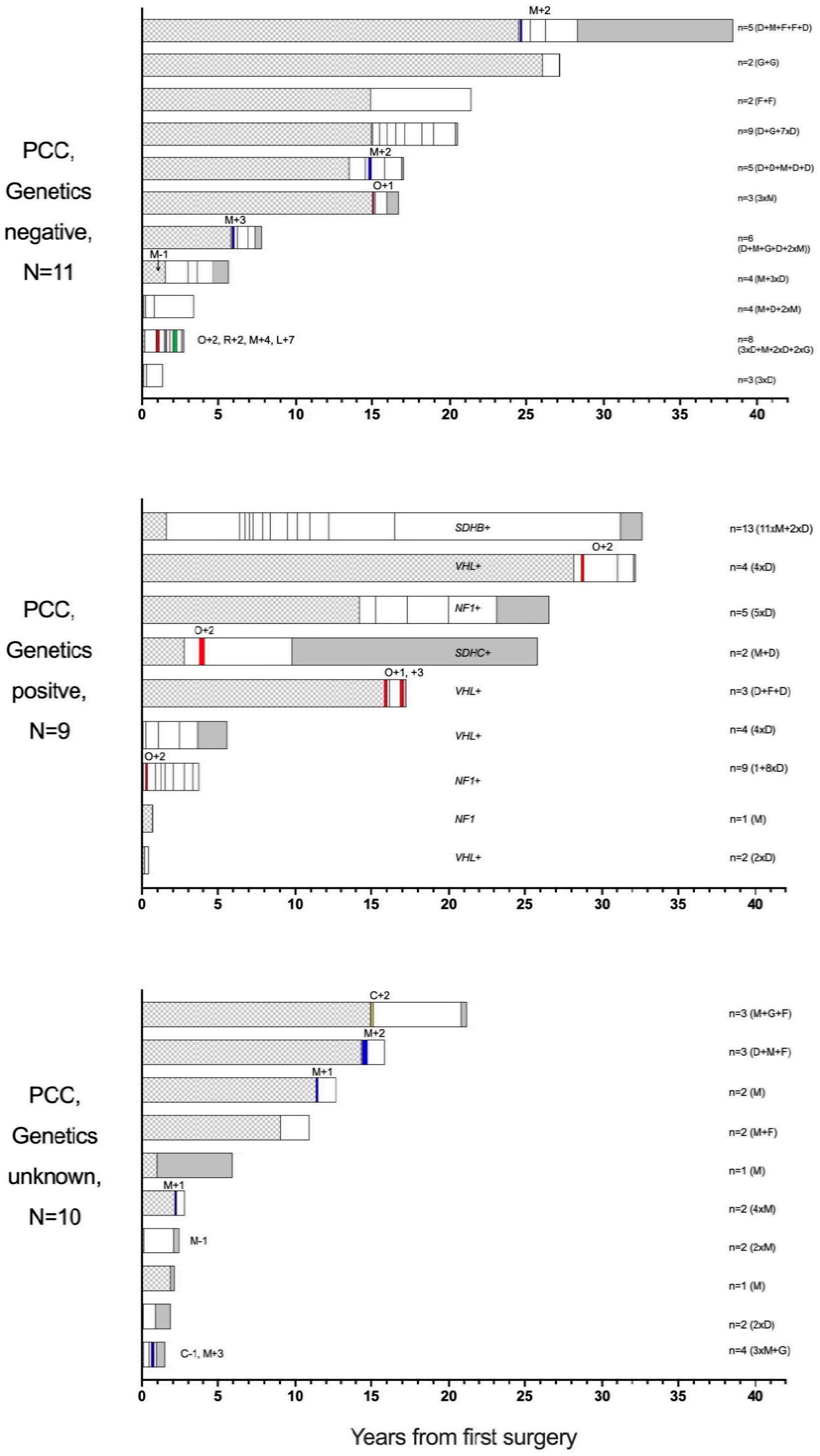

Supplementary Figure S1a

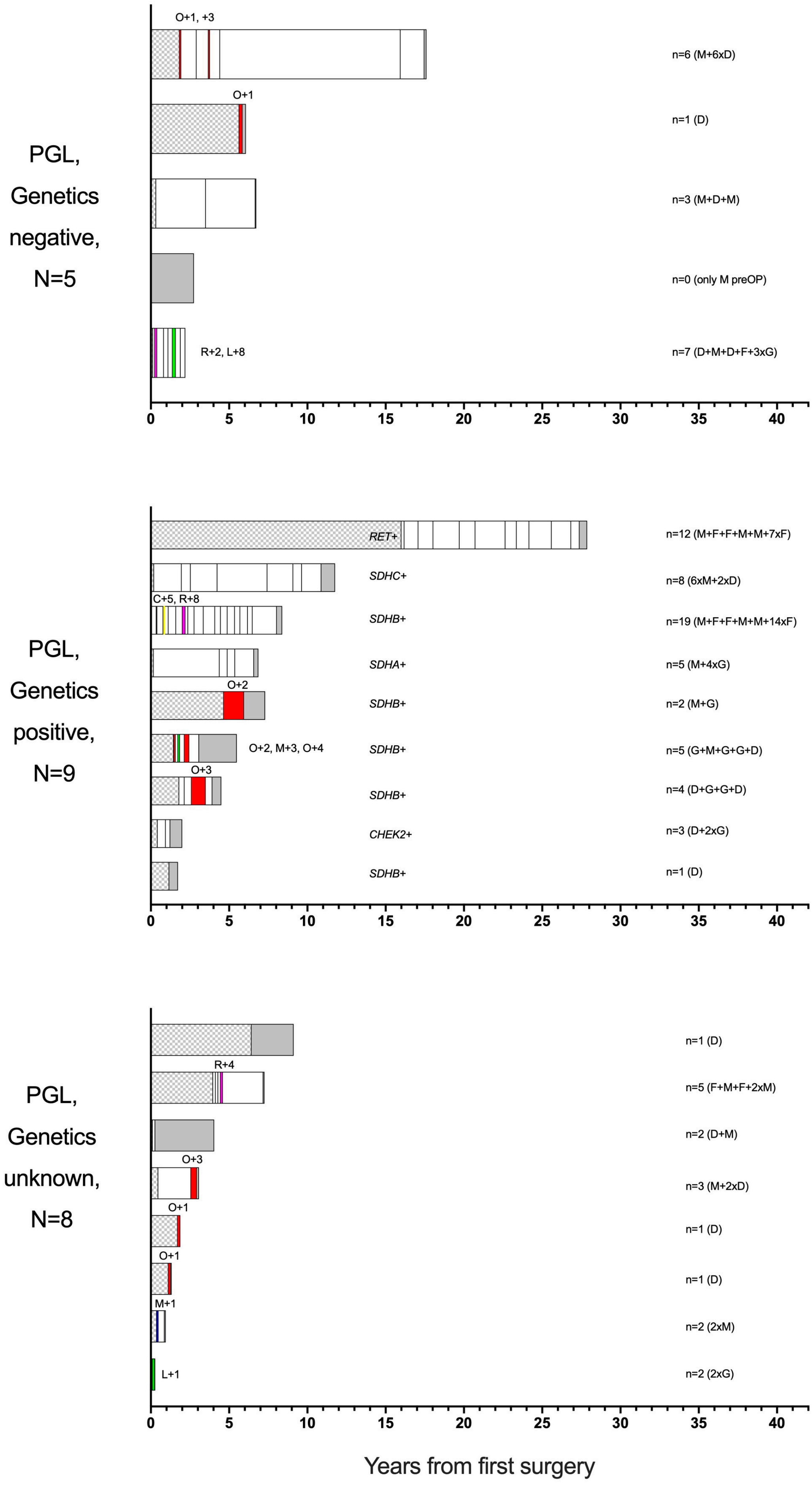

Supplementary Figure S1b

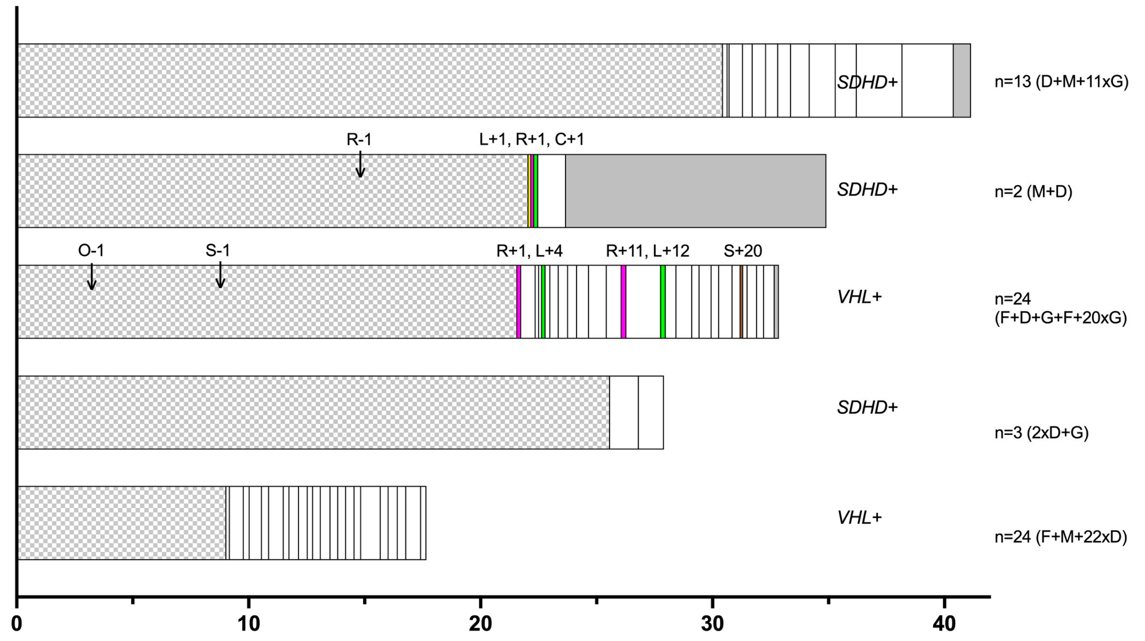

Supplementary Figure S1c

Supplementary Figure S1. Details and temporal relationship of imaging studies of 30 patients with metastatic PCC (Supplementary Figure S1a), of 22 with metastatic PGL (Supplementary Figure S1b) and of 5 with metastatic HNPGL (Supplementary Figure S1c) divided by germline genetic results. The total FU after first surgery is given on the x-axis, individual patients in order of increasing FU duration on the y-axis. The number and modalities of all imaging studies (M=^{123}MIBG, D=^{18}F-DOPA, F=^{18}FDG, G=^{68}GaDOTA) are depicted to the right of every horizontal bar for every patient, the specific gene in those with pathogenic germline variants to the left thereof. The light grey coloured parts of the bars on the far left represent the time from first surgery to the first FU imaging study, the dark grey coloured part at the far right the time from the last FU imaging to the end of FU. Widths of the bar sections indicate the time from one imaging study to the next. Therapies during FU (C=systemic chemotherapy, yellow; L=^{177}Lutetium-therapy, green; M=^{131}MIBG-therapy, blue; O=operation, red; R=external radiation, magenta; S=SIRT [selective intraarterial embolization therapy) are given at appropriate times (-1 indicating before first surgery, +1, +2,… after first FU imaging study, after the second,…) above the respective time of FU, the width of the coloured section the time elapsed since the preceding imaging study.
